# Update on the global burden of acute viral hepatitis in 2021: addressing health inequalities

**DOI:** 10.3389/fpubh.2025.1580863

**Published:** 2025-05-23

**Authors:** Lei Zhang, Ting Wang, Shan Zhou, Shengpeng Li, Ting Mo, Shuanghua Wu

**Affiliations:** ^1^The Friendship Hospital of Yili Kazakh Autonomous Prefecture, Yining, China; ^2^Department of Rehabilitation Medicine, Zhuzhou Hospital Affiliated to Xiangya School of Medicine, Central South University, Zhuzhou, China; ^3^Department of Critical Care Medicine, Zhuzhou Hospital Affiliated to Xiangya School of Medicine, Central South University, Zhuzhou, China

**Keywords:** acute viral hepatitis, global burden of disease, health equity, incidence, sociodemographic index

## Abstract

**Background:**

The strategy for eliminating viral hepatitis is at a critical juncture, necessitating an updated assessment of global incidence trends.

**Methods:**

Data on the incidence of four types of acute viral hepatitis (AVH), namely, acute hepatitis A (AHA), acute hepatitis B (AHB), acute hepatitis C (AHC), and acute hepatitis E (AHE), were sourced from the Global Burden of Disease (GBD) Study 2021. The annual percentage change is utilized to elucidate temporal trends, whereas health inequalities and frontier analysis serve to evaluate global health equity and quantify disparities in burden among countries.

**Results:**

In 2021, the estimated global incidence for AVH was as follows: AHA (160.86 million), AHB (63.53 million), AHE (19.37 million), and AHC (7.01 million). From 2000 to 2021, the age-standardized incidence rates (ASIR) for four types of AVH demonstrated a declining trend, with AHB showing the most significant decrease. It is anticipated that the incidence rates for AHA, AHB, and AHC will continue to decline over the next 15 years; however, the incidence rate of AHE is projected to increase. In 2021, the incidence of AVH displayed a significant negative correlation with the Socio-Demographic Index (SDI), but health disparities between countries have diminished. Countries with the highest potential for elimination of AHB are primarily situated in low and low-middle SDI regions, whereas those for AHA are concentrated in high and high-middle SDI regions. Furthermore, countries with the largest disparities in AHC and AHE were dispersed.

**Conclusion:**

Although global incidence of AVH is decreasing, it remains a serious public health challenge. Reducing health disparities is crucial for the elimination of viral hepatitis.

## Introduction

Acute viral hepatitis remains a significant global public health threat, with new infections and the progression to chronicity continuing unabated. Reports indicate that in 2019, there were 263 million new cases of various types of acute viral hepatitis globally, despite an overall declining trend in the incidence of acute viral hepatitis (AVH) (1). Viral hepatitis can be classified into five main types (A-E), each with distinct transmission mechanisms and risks of chronicity (2). Acute hepatitis A (AHA) and acute hepatitis E (AHE) are primarily transmitted via the fecal-oral route and typically present as acute self-limiting hepatitis (3). Although the probability of acute hepatitis B (AHB) developing into chronic infection is very low in healthy adults, it reaches 30% in children under 6 years old and exceeds 80% in newborns through mother-to-child transmission (4). Similarly, without intervention, approximately 80% of acute hepatitis C (AHC) cases will progress to chronic infection (lasting more than 6 months) (5, 6). Hepatitis D occurs only in conjunction with hepatitis B virus infection, exacerbating liver damage (7).

Chronic viral hepatitis is the leading cause of liver cirrhosis and liver cancer, affecting around 300 million people globally and resulting in 1.3 million deaths (8). Notably, effective vaccines are now available for hepatitis A (HAV), hepatitis B (HBV), and hepatitis E (HEV). The HBV vaccine has been widely implemented in infant vaccination programs worldwide (9). As of February 2023, a total of 59 countries have included the HAV vaccine in their immunization schedules or targeted high-risk populations (10). However, the HEV vaccine is approved for use in only a few countries, such as China and Pakistan (11, 12). In recent years, antiviral treatment strategies for chronic hepatitis have rapidly evolved, particularly with the advent of direct-acting antiviral agents (DAAs) offering hope for the elimination of hepatitis C virus (HCV) (13, 14). Against this backdrop, the World Health Organization’s goal to eliminate hepatitis by 2030 aims to reduce new chronic infections by 90% and mortality by 65% from 2016 to 2030 (15). However, achieving this goal is exceedingly difficult in low-and middle-income regions and certain high-risk countries due to disparities in socioeconomic status, vaccination rates, and coverage of antiviral treatments (16, 17).

The Global Burden of Disease (GBD) Study evaluates the disease burden across 204 countries and regions, providing a robust data foundation for understanding the global epidemiological trends of acute viral hepatitis (18). Previous research has indicated a notable decline in the disability-adjusted life years attributed to acute viral hepatitis, which is inversely correlated with Socio-Demographic Index (SDI) (1, 19). This study aims to utilize GBD 2021 data to report the latest estimates of global acute viral hepatitis incidence and trends, assess health inequalities in the burden of acute viral hepatitis across countries with varying SDI levels, and project future trends. This research serves as a reference for enhancing global health equity and optimizing medical resource allocation strategies necessary for achieving the goal of eliminating viral hepatitis.

## Methods

### Data sources

The data for this study were obtained from GBD 2021, which is publicly accessible through the online platform maintained by the Institute for Health Metrics and Evaluation.^1^ The study extracted incidence data for four types of acute viral hepatitis (AHA, AHB, AHC, AHE) from 2000 to 2021. Acute Hepatitis D (AHD) is not included in the GBD 2021 dataset and, therefore, is excluded from this analysis. Additionally, the study examines various factors, including geographical regions (global, 21 regions, and 204 countries and territories), gender, age, and levels of SDI. Detailed methodologies are provided in the Supplementary methods.

### Assessment metrics

The general estimation methodology employed in GBD 2021 has been described in depth elsewhere (18). The incidence data included the number of cases, crude incidence rates, and age-standardized incidence rates (ASIR). Case numbers are reported as total incidence for each year, accompanied by the 95% uncertainty interval (UI); rates are presented as the number of cases per 100,000 population, also with a 95% UI.

### Health inequality and frontier analysis

In accordance with WHO recommendations, the slope index of inequality (SII) and concentration index (CI) were utilized to measure absolute and relative health inequalities between different countries (20, 21). The SII reflects the absolute health disparities between impoverished and affluent groups by fitting a linear relationship between health indicators and SDI levels. The CI ranges from −1 to 1, with values greater than zero indicating superior health status in affluent groups, while values less than zero indicate better health in poorer groups. An absolute CI value closer to zero signifies a smaller relative health disparity. Frontier analysis was utilized to evaluate the minimum achievable burden of acute viral hepatitis (AVH) incidence in countries at various SDI levels (22). Data Envelopment Analysis (DEA) and Locally Estimated Scatterplot Smoothing (LOESS) methods were applied to fit frontier boundary curves, and the distances from these boundaries were used to quantify effective disparities among countries after adjusting for SDI levels.

### Data statistics and analysis

The Joinpoint Regression Program (version 5.1.0.0) was employed to calculate annual percentage changes (APC) to evaluate average temporal trends in ASIR (23, 24). An APC and its corresponding 95% confidence intervals greater or less than zero indicate an increasing or decreasing trend in ASR, respectively. If the confidence interval includes zero, the trend is considered statistically insignificant. Spearman correlation tests were conducted to assess relationships between different indicators and SDI levels (25). The Bayesian Age-Period-Cohort (BAPC) model was utilized to predict global incidence trends of acute viral hepatitis over the next 15 years (26). In addition to APC calculations, all other statistical analyses and visualizations were performed using R (version 4.4.0) with packages including mgcv (version 1.9–1), ggplot2 (version 3.5.1), INLA (version 23.06.29), and BAPC (version 0.0.36).

## Results

### Incidence trends of acute hepatitis A

**Figure 1:**
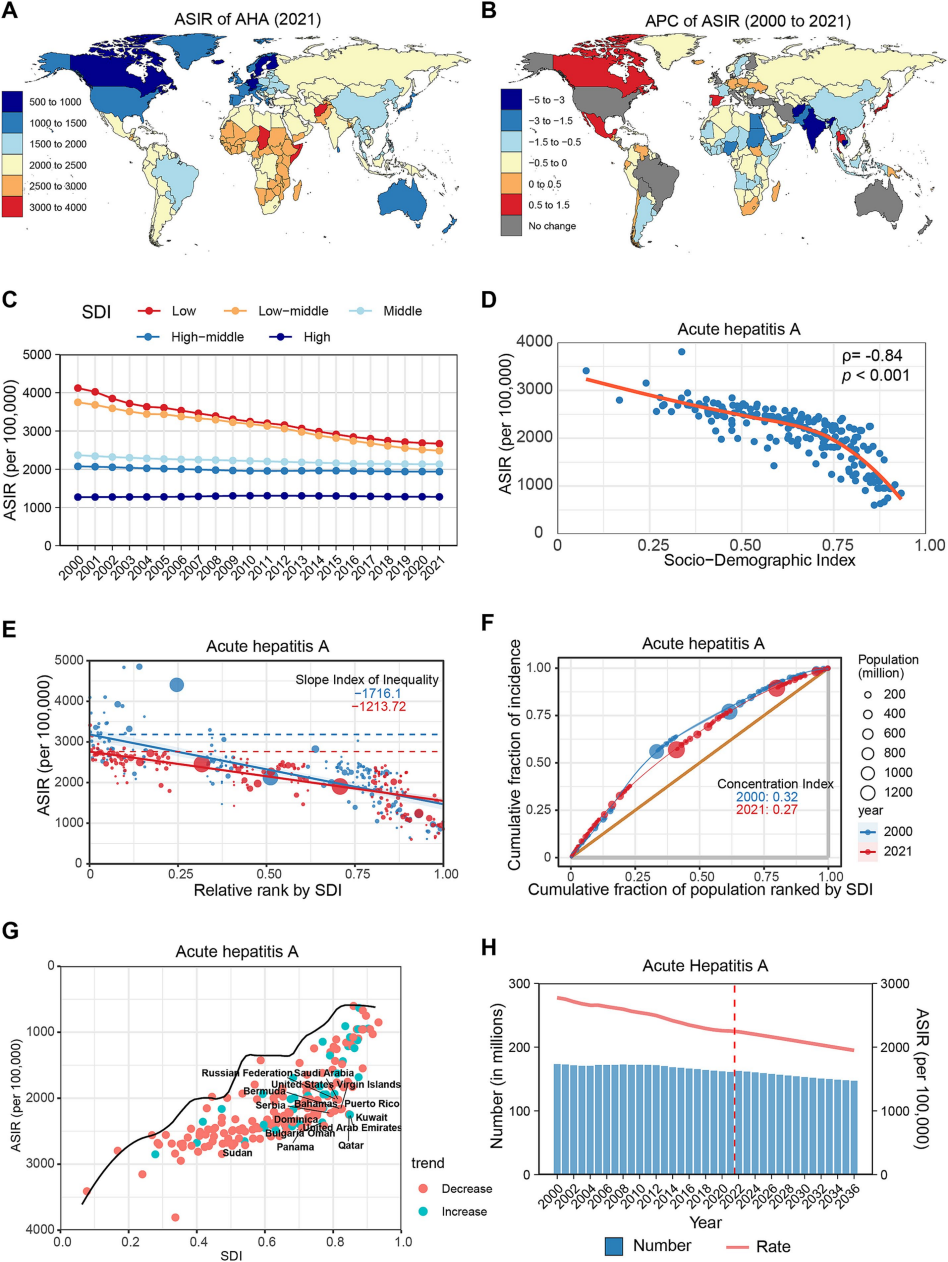
Global trends in the incidence of AHA. **(A)** ASIR of AHA in 204 countries and regions in 2021. **(B)** APC of AHA in 204 countries and regions from 2000 to 2021. **(C)** Changes in the incidence of AHA across five categories of SDI regions. **(D)** The relationship between AHA incidence and SDI levels in 204 countries and regions. Health inequality analyses reveal SII **(E)** and CI **(F)**. **(G)** Frontier analyses identify the 10 countries with the largest burden disparities of AHA after adjusting for SDI levels. **(H)** Predictions of AHA incidence changes over the next 15 years using the BAPC model. AHA, acute hepatitis A; ASIR, Age-standardized incidence rates; APC, Annual percentage change; SDI, Sociodemographic Index; SII, Slope Index of Inequality; CI, concentration index; BAPC, Bayesian age-period-cohort.

In 2021, AHA emerged as the most prevalent type of AVH, accounting for 64.15% of all new infections, with an estimated 160.86 (95% UI: 152.2–170.43) million new cases globally. The ASIR was calculated at 2273.72 (95% UI: 2150.13–2403.76) per 100,000 population (Table 1). Regionally, the highest ASIRs were observed in Eastern Sub-Saharan Africa (2732.42), Western Sub-Saharan Africa (2582.89), and Southern Sub-Saharan Africa (2518.31) (Supplementary Figure S1A). At the country level, Afghanistan (3807.66), Somalia (3956.15), and Chad (3153.37) exhibited the highest ASIRs (Figure 1A). No significant gender differences were noted among new AHA cases in 2021, with the highest incidence reported in children under 5 years of age, decreasing consistently with age (Supplementary Figure S2A).

**Table 1:** Global incidence number and ASIR of acute hepatitis, from 2000 to 2021.

Types	Number of incidence, No. × 10^6^ (95% UI)		ASIR per 100,000, No. (95% UI)		APC of ASIR
	2000	2021	2000	2021	2000 to 2021
Acute hepatitis A					
Both	173.11 (158.83, 190.07)	160.86 (152.2, 170.43)	2784.64 (2549.94, 3070.47)	2273.72 (2150.13, 2403.76)	−1.02 (−1.08, −0.97)
Male	88.75 (81.24, 97.7)	82.47 (78.02, 87.1)	2783.93 (2537.76, 3078.17)	2271.19 (2148.62, 2400.95)	−0.99 (−1.03, −0.94)
Female	84.36 (77.64, 92.34)	78.39 (74.08, 83.22)	2787.31 (2562.77, 3061.29)	2276.47 (2152.07, 2412.4)	−1.07 (−1.14, −1.00)
Acute hepatitis B					
Both	76.26 (62.49, 92.38)	63.53 (50.45, 78.88)	1224.72 (1004.81, 1478.76)	784.73 (627.37, 968.45)	−2.07 (−2.10, −2.03)
Male	46.94 (37.77, 58.16)	38.98 (30.96, 49.53)	1499.45 (1214.22, 1852.05)	957.85 (765.08, 1212.49)	−2.03 (−2.09, −1.97)
Female	29.31 (22.32, 37.74)	24.56 (18.78, 32.16)	948.7 (720.7, 1215.31)	611.59 (468.2, 798.5)	−2.11 (−2.16, −2.07)
Acute hepatitis C					
Both	5.67 (5.02, 6.39)	7.01 (6.18, 7.89)	96.63 (85.77, 108.33)	92.64 (82.13, 104.65)	−0.33 (−0.39, −0.26)
Male	2.87 (2.54, 3.23)	3.55 (3.14, 4.01)	97.36 (86.46, 109.02)	93.66 (82.89, 105.57)	−0.28 (−0.35, −0.21)
Female	2.8 (2.48, 3.16)	3.46 (3.05, 3.89)	96.04 (84.9, 107.85)	91.69 (81.15, 103.84)	−0.38 (−0.46, −0.30)
Acute hepatitis E					
Both	17.09 (14.17, 20.52)	19.37 (16.1, 23.24)	269.62 (224.77, 320.87)	260.41 (215.39, 312.21)	−0.20 (−0.23, −0.18)
Male	8.83 (7.29, 10.64)	9.93 (8.26, 11.94)	273.08 (227.49, 324.86)	262.57 (217.26, 315.49)	−0.23 (−0.26, −0.20)
Female	8.26 (6.87, 9.91)	9.44 (7.86, 11.32)	265.95 (222.39, 317.31)	258.11 (213.68, 309.09)	−0.18 (−0.19, −0.16)

ASIR, age-standardized incidence rate; APC, annual percent change; UI, uncertainty interval.

From 2000 to 2021, the ASIR for AHA demonstrated an overall declining trend globally (APC: −1.02, 95% CI: −1.08 to −0.97) (Table 1). Most regions reported a decrease in ASIR, with the most significant declines observed in South Asia (APC: −2.62%), Western Sub-Saharan Africa (APC: −1.36%), and North Africa and the Middle East (APC: −0.88%). However, Central Latin America (APC: 0.26%), Oceania (APC: 0.24%), and High-Income North America (APC: 0.15%) experienced slight increases (Supplementary Figure S1B; Supplementary Table S1). At the national level, 150 countries and regions demonstrated a declining trend, with the most significant decreases occurring in Afghanistan (APC: −4.35%), Cambodia (APC: −3.12%), and India (APC: −3.09%). Conversely, 32 countries and regions exhibited an increasing trend, with the most notable increases recorded in Canada (APC: 1.25%), Thailand (APC: 1.1%), and Japan (APC: 0.89%). The remaining countries showed no significant change (Figure 1B; Supplementary Table S2).

In 2021, within the SDI classification, the ASIR of AHA was highest in the low SDI group and progressively decreased as SDI levels increased. From 2000 to 2021, the most pronounced decline was observed in low-middle SDI regions (APC: −2.05, 95% CI: −2.16 to −1.94), while high SDI regions remained stable (APC: 0.06, 95% CI: −0.01 to 0.13) (Figure 1C; Supplementary Table S4). Spearman correlation analysis revealed a significant negative correlation between AHA incidence and SDI level (*ρ* = −0.84, *p* < 0.001) (Figure 1D). Furthermore, the study identified significant absolute and relative health inequalities in AHA incidence among different countries (Figures 1E,F). The Slope Index of Inequality (SII) in 2000 was −1716.1, indicating that countries with the lowest SDI experienced an additional 1716.1 cases per 100,000 population compared to those with the highest SDI; this gap decreased to 1213.72 cases per 100,000 population by 2021. Similarly, the Concentration Index (CI) declined from 0.32 to 0.27. After adjusting for SDI levels, countries with the largest disparities in AHA incidence compared to the frontier level are concentrated in high and high-middle SDI countries, such as Qatar, the United Arab Emirates, and Kuwait (Figure 1G; Supplementary Table S5). Projections suggest that by 2030, the global number of AHA cases could decrease to 152.9 million, with an ASIR of 2074.9 per 100,000 population (Figure 1H; Supplementary Table S6).

### Incidence trends of acute hepatitis B

**Figure 2:**
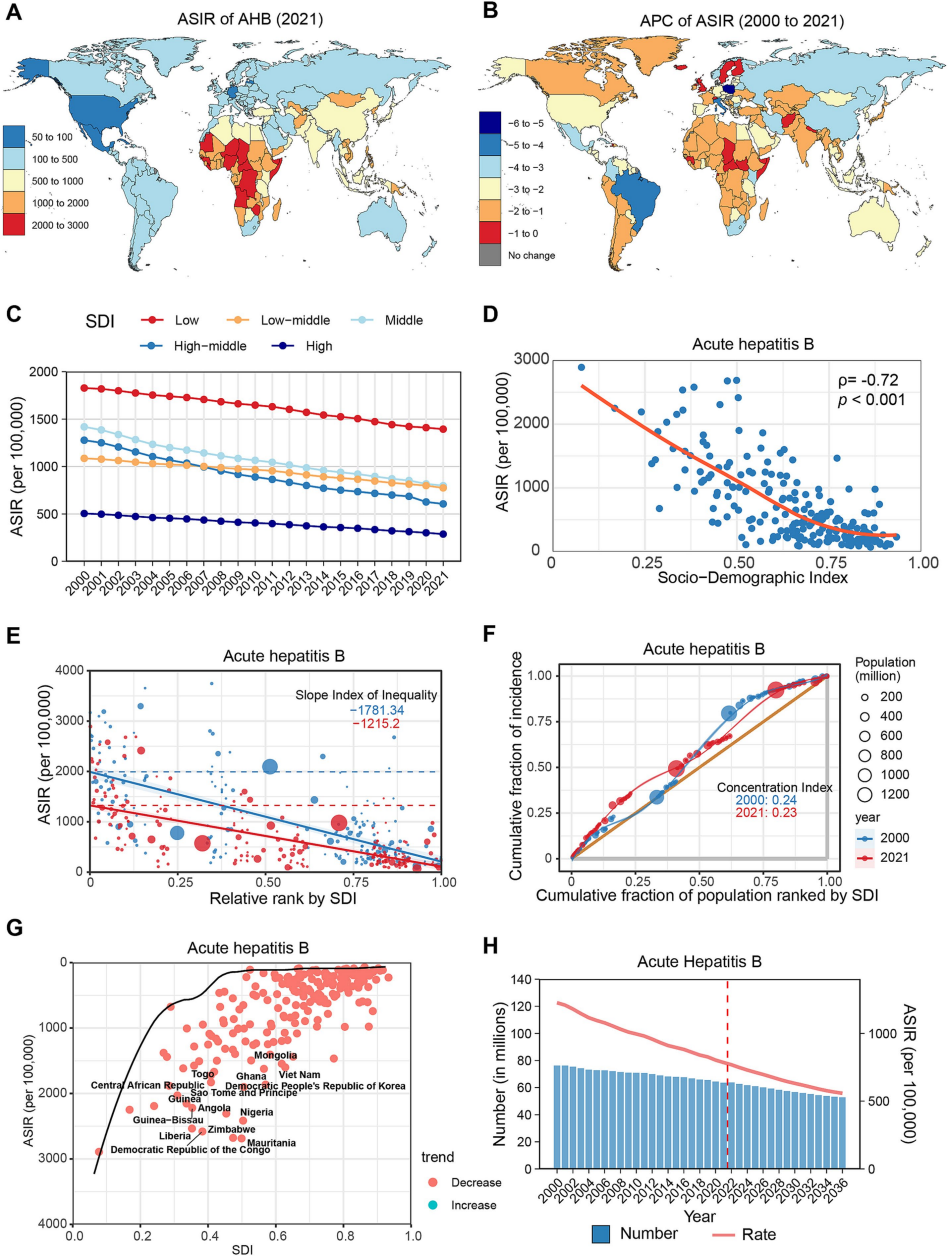
Global trends in the incidence of AHB. **(A)** ASIR of AHB in 204 countries and regions in 2021. **(B)** APC of AHB in 204 countries and regions from 2000 to 2021. **(C)** Changes in the incidence of AHB across five categories of SDI regions. **(D)** The relationship between AHB incidence and SDI levels in 204 countries and regions. Health inequality analyses reveal SII **(E)** and CI **(F)**. **(G)** Frontier analyses identify the 10 countries with the largest burden disparities of AHB after adjusting for SDI levels. **(H)** Predictions of AHB incidence changes over the next 15 years using the BAPC model. AHB, acute hepatitis B; ASIR, Age-standardized incidence rates; APC, Annual percentage change; SDI, Sociodemographic Index; SII, Slope Index of Inequality; CI, concentration index; BAPC, Bayesian age-period-cohort.

In 2021, AHB ranked second among AVH types, with an estimated 63.53 (95% UI: 50.45–78.88) million new infections globally and an ASIR of 784.73 (95% UI: 627.37–968.45) per 100,000 population (Table 1). At the regional level, the highest ASIRs were recorded in Central Sub-Saharan Africa (2405.86), Western Sub-Saharan Africa (2065.91), and Oceania (1522.89) (Supplementary Figure S1A). By country, Somalia (2891.48), Mauritania (2686.97), and Zimbabwe (2677.96) exhibited the highest ASIRs (Figure 2A). Additionally, in 2021, the ASIR for males was approximately 1.57 times that of females, peaking in the 30–34 age group and declining on either side (Supplementary Figure S2B).

From 2000 to 2021, the ASIR of AHB displayed a downward trend globally (APC: −2.07, 95% CI: −2.10 to −2.03) (Table 1). All regions experienced a decline, with the most significant reductions noted in Tropical Latin America (APC: −4.19%), East Asia (APC: −3.42%), and Eastern Europe (APC: −3.15%) (Supplementary Figure S1B; Supplementary Table S1). At the country level, all except Hungary and Denmark displayed a declining trend, with the most notable decreases recorded in Poland (APC: −5.12%), Italy (APC: −4.57%), and Brazil (APC: −4.25%) (Figure 2B; Supplementary Table S2). Notably, incidence rates decreased across all age groups, particularly among those under 15 years. During the study period, AHB cases decreased by 58.18% among children under 5 years, with their total proportion declining from 6.54 to 3.28% (Supplementary Figure S2E; Supplementary Table S3).

In 2021, the ASIR of AHB was highest in low SDI regions, followed by middle, lower-middle, high-middle, and high SDI regions. From 2000 to 2021, the high-middle SDI regions experienced the greatest decrease (APC: −3.43, 95% CI: −3.53 to −3.33), while the low SDI regions had the smallest decrease (APC: −1.33, 95% CI: −1.40 to −1.27) (Figure 2C; Supplementary Table S4). A significant negative correlation existed between AHB incidence and SDI levels (*ρ* = −0.72, *p* < 0.001) (Figure 2D). Health inequalities among countries diminished during the study period, evidenced by a decrease in SII from −1781.34 to −1215.2 and in CI from 0.24 to 0.23 (Figures 2E,F). Frontier analysis indicates that countries with the largest burden disparities in AHB are concentrated in low and low-middle SDI regions, notably Mauritania, Zimbabwe, and Nigeria (Figure 2G; Supplementary Table S5). Projections indicate that by 2030, the global number of AHB cases will decrease to 56.66 million, with an ASIR of 631.2 per 100,000 population (Figure 2H; Supplementary Table S6).

### Incidence trends of acute hepatitis C

**Figure 3:**
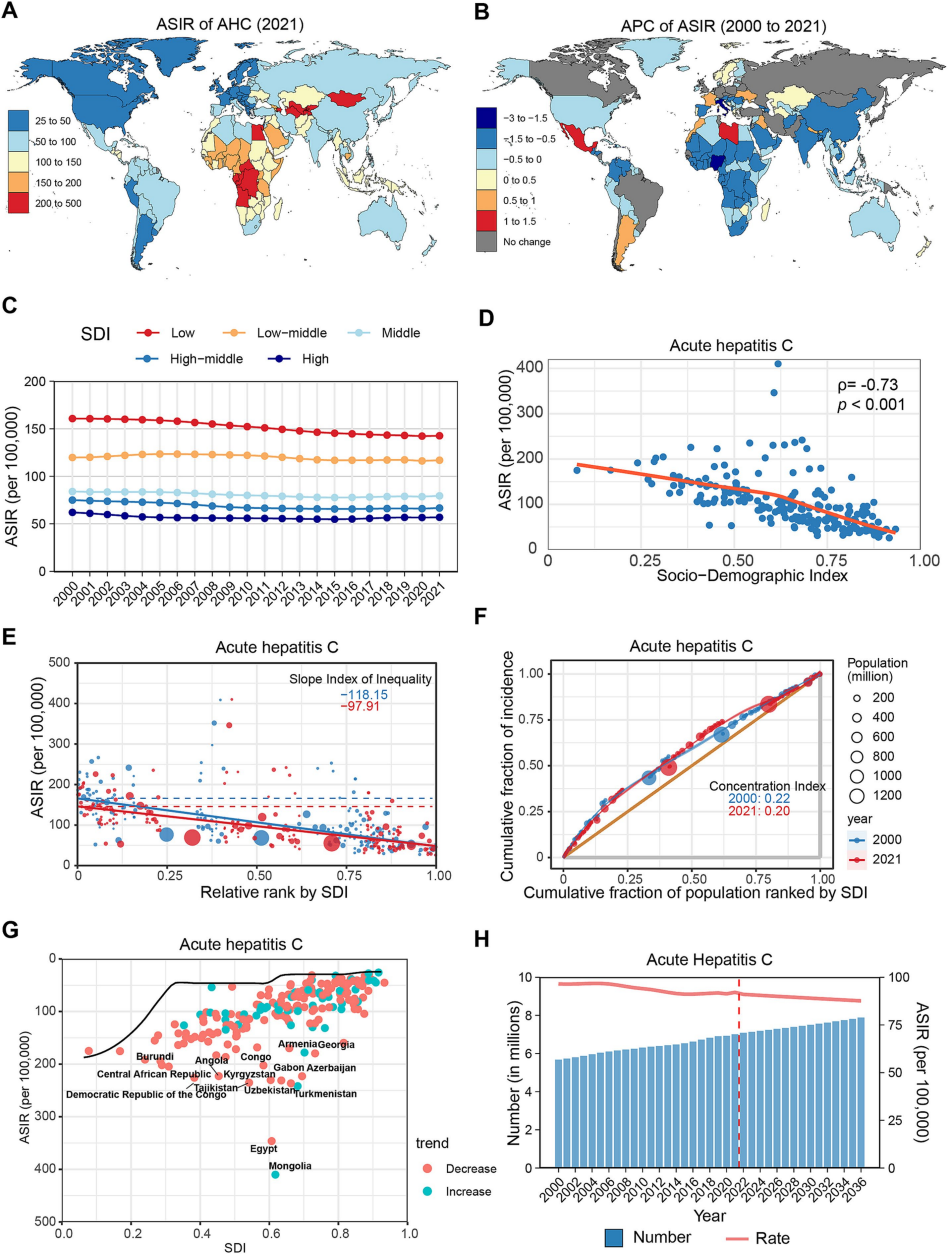
Global trends in the incidence of AHC. **(A)** ASIR of AHC in 204 countries and regions in 2021. **(B)** APC of AHC in 204 countries and regions from 2000 to 2021. **(C)** Changes in the incidence of AHC across five categories of SDI regions. **(D)** The relationship between AHC incidence and SDI levels in 204 countries and regions. Health inequality analyses reveal SII **(E)** and CI **(F)**. **(G)** Frontier analyses identify the 10 countries with the largest burden disparities of AHC after adjusting for SDI levels. **(H)** Predictions of AHC incidence changes over the next 15 years using the BAPC model. AHC, acute hepatitis C; ASIR, Age-standardized incidence rates; APC, Annual percentage change; SDI, Sociodemographic Index; SII, Slope Index of Inequality; CI, concentration index; BAPC, Bayesian age-period-cohort.

In 2021, AHC accounted for the smallest share among the four types of AVH, with approximately 7.01 (95% UI: 6.18–7.89) million new cases globally and an ASIR of 92.64 (95% UI: 82.13–104.65) per 100,000 population (Table 1). The highest ASIRs were reported in Central Sub-Saharan Africa (222.72), Central Asia (218.31), and Western Sub-Saharan Africa (166.07) (Supplementary Figure S1A). At the country level, Mongolia (410.06), Egypt (346.24), and Turkmenistan (241.84) displayed the highest ASIRs, with Egypt’s high estimates likely linked to extensive HCV screening programs (Figure 3A). There were no significant gender disparities in AHC incidence, but a notable majority of new cases occurred in children under 5 years, showing a decreasing trend until the 20–24 age group, after which rates increased with age (Supplementary Figure S2C).

From 2000 to 2021, the global ASIR of AHC only slightly decreased (APC: −0.33, 95% CI: −0.39 to −0.26), despite an increase in the overall incidence number by 23.54% (Table 1). Most regions exhibited a declining trend, except for Southern Latin America (APC: 0.31%), Oceania (APC: 0.17%), and Central Latin America (APC: 0.17%), which experienced slight increases (Supplementary Figure S1B; Supplementary Table S1). At the country level, 39 countries showed increased AHC incidence, notably Libya (APC: 1.42%), Mexico (APC: 1.16%), and Morocco (APC: 0.96%). Conversely, 136 countries reported declines, with significant reductions in Italy (APC: −2.68%), Nigeria (APC: −1.85%), and Gabon (APC: −1.37%) (Figure 3B; Supplementary Table S2). Age-wise, the ASIR for AHC showed a decreasing trend among older adults (>50 years), while trends in younger populations (<50 years) remained stable or slightly increased (Supplementary Figure S2E; Supplementary Table S3).

In the SDI classification, AHC incidence consistently decreased with increasing SDI levels (Figure 3C; Supplementary Table S4). Throughout the study period, all five SDI regions recorded declines, with the low SDI region exhibiting the greatest decrease (APC: −0.69, 95% CI: −0.75 to −0.64). Spearman correlation analysis indicated a significant negative correlation between AHC incidence and SDI level (*ρ* = −0.73, *p* < 0.001) (Figure 3D). Furthermore, health inequality analysis indicated that the SII reduced from −118.15 to −97.91, while the CI decreased from 0.22 to 0.20 (Figures 3E,F). Frontier analysis reveals that countries exhibiting the greatest disparities in new HCV infections are distributed across the SDI classification, with Mongolia, Egypt, and Turkmenistan being the most notable examples (Figure 3G; Supplementary Table S5). Projections suggest that by 2030, the global incidence of AHC will surpass 7.5 million, with an ASIR of 89.02 per 100,000 population (Figure 3H; Supplementary Table S6).

### Incidence trends of acute hepatitis E

**Figure 4:**
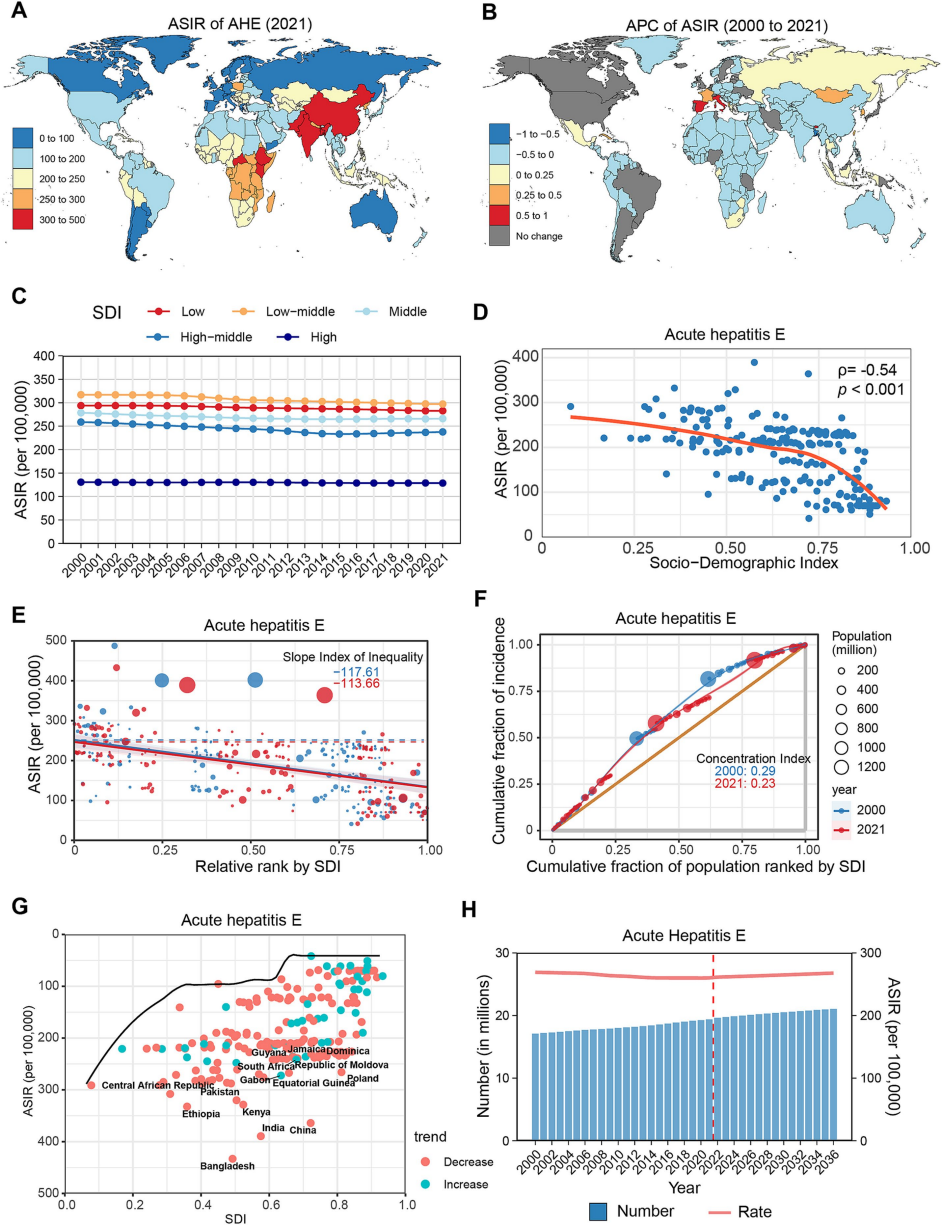
Global trends in the incidence of AHE. **(A)** ASIR of AHE in 204 countries and regions in 2021. **(B)** APC of AHE in 204 countries and regions from 2000 to 2021. **(C)** Changes in the incidence of AHE across five categories of SDI regions. **(D)** The relationship between AHE incidence and SDI levels in 204 countries and regions. Health inequality analyses reveal SII **(E)** and CI **(F)**. **(G)** Frontier analyses identify the 10 countries with the largest burden disparities of AHE after adjusting for SDI levels. **(H)** Predictions of AHE incidence changes over the next 15 years using the BAPC model. AHE, acute hepatitis E; ASIR, Age-standardized incidence rates; APC, Annual percentage change; SDI, Sociodemographic Index; SII, Slope Index of Inequality; CI, concentration index; BAPC, Bayesian age-period-cohort.

In 2021, it was estimated that there were 19.37 (95% UI: 16.1–23.24) million new cases of AHE globally, with an ASIR of 260.41 (95% UI: 215.39–312.21) per 100,000 population (Table 1). Regionally, the highest ASIRs were recorded in South Asia (380.41), East Asia (360.02), and Eastern Sub-Saharan Africa (294.69) (Supplementary Figure S1A). At the country level, the highest ASIRs were in Bangladesh (433.01), India (389.37), and China (363.96) (Figure 4A). No significant gender differences were evident in the ASIR; however, the highest incidence rates were found among individuals aged 5–9 years, decreasing until the lowest rates were seen in the 55–59 age group, after which rates increased with age (Supplementary Figure S2D).

From 2000 to 2021, the global number of AHE cases increased by 13.37%, while the ASIR slightly decreased (APC: −0.20, 95% CI: −0.23 to −0.18) (Table 1). Regionally, ASIRs increased in Western Europe (APC: 0.26%), High-Income Asia Pacific (APC: 0.17%), and Southern Sub-Saharan Africa (APC: 0.16%). In contrast, East Asia (APC: −0.46%), South Asia (APC: −0.29%), and North Africa and the Middle East (APC: −0.19%) displayed the most significant declines (Supplementary Figure S1B; Supplementary Table S1). At the country level, AHE incidence increased in 18 countries, with notable increases in Spain (APC: 0.99%), Bhutan (APC: 0.82%), and Italy (APC: 0.71%). Conversely, 157 countries experienced declines, with the most significant reductions observed in Bangladesh (APC: −0.78%), China (APC: −0.49%), and Türkiye (APC: −0.33%) (Figure 4B; Supplementary Table S2). During the study period, AHE rates increased among adults over 40 years, while rates among those under 40 diminished (Supplementary Figure S2E; Supplementary Table S3).

In the SDI subgroup analysis, the ASIR of AHE in 2021 was highest in the lower-middle SDI group. Throughout the study period, all five SDI regions reported decreases, particularly the upper-middle SDI regions, which experienced the most substantial decline (APC: −0.51, 95% CI: −0.60 to −0.42) (Figure 4C; Supplementary Table S4). A negative correlation was identified between AHE incidence and SDI levels (*ρ* = −0.54, *p* < 0.001) (Figure 4D). Health inequality analysis demonstrated a reduction in SII from −117.61 to −113.66, while the CI decreased from 0.29 to 0.23 (Figures 4E,F). Frontier analysis reveals that countries exhibiting the largest gaps from the frontier are distributed across the SDI classification, with Bangladesh, China, and India representing the most significant examples (Figure 4G; Supplementary Table S5). Projections indicate that by 2030, the global number of AHE cases will rise to 20.5 million, with an ASIR of 264.77 per 100,000 population (Figure 4H; Supplementary Table S6).

## Discussion

This study, utilizing data from the GBD 2021, presents an updated analysis of the global burden of AVH and trends in the incidence rates of its four major types from 2000 to 2021. It emphasizes health inequalities associated with AVH burdens across different countries and identifies those with significant potential for eliminating various AVH types.

In 2021, AHA had the highest estimated incidence of AVH globally, followed by AHB, AHE, and AHC. While all AVH types exhibited declining ASIR, there was an increase in new cases of AHC and AHE, likely attributable to population growth and enhanced screening efforts. Contrary to previous studies, our findings revealed that, aside from the male predominance in AHB, no significant gender differences were observed in other AVH types (19). Additionally, the ASIR for four AVH types negatively correlated with SDI, highlighting the need for increased attention to the AVH burden in low and middle-low SDI regions (27). Notably, some alleviation of health inequalities among countries was observed during the study period, fostering optimism for advancing global health equity.

AHA remains the most common type of AVH globally, primarily transmitted through contaminated water or food (28). Its incidence has been declining worldwide, particularly in low and low-middle SDI regions, attributed to improvements in public health and the implementation of Hepatitis A vaccination programs. GBD 2021 data indicate that new AHA infections are concentrated in low-and middle-income countries, notably Afghanistan, while high-income regions such as Qatar exhibit significant potential for AHA elimination. As of 2021, over 40% of new AHA cases occurred in children under 5 years old, underscoring the urgent need to expand coverage for Hepatitis A vaccination. Immunization with two doses of the Hepatitis A vaccine can typically provide protection for over 15 years (29, 30). Furthermore, the WHO reports that a single-dose regimen among children can achieve immunity equivalent to that of a two-dose regimen, proving more cost-effective and easier to implement (31, 32).

Conversely, AHE, also a fecal-oral infectious disease (33), has seen an increase in cases globally, presenting new epidemiological characteristics. HEV genotypes 1 and 2 primarily affect low-income countries and are transmitted through the fecal-oral route, while genotypes 3 and 4, which exhibit zoonotic transmission, have become increasingly prevalent in developed countries (34, 35). With advancements in public health, the composition of AHE cases may shift toward HEV genotypes 3 and 4, as observed in China, where new infections of HEV genotype 4 have already surpassed those of genotype 1 (36). Our research indicates that, in 2021, new HEV infections were concentrated in children and adolescents. Additional studies have demonstrated elevated HEV infection rates among pregnant women in high-prevalence areas, correlating with increased maternal mortality and adverse fetal outcomes (37). Recent clinical trials indicate that vaccination with a three-dose HEV vaccine can confer protection for up to 10 years in adults (38). While HEV vaccines have shown efficacy and tolerability in non-pregnant populations, caution is necessary as they may increase the risk of miscarriage in pregnant women (39). Thus, further evidence supporting the safety of HEV vaccines among children and pregnant women (40), along with comparative studies on dosing, is essential to achieving global AHE elimination.

Chronic infections resulting from AHB and AHC are major contributors to viral hepatitis-related mortality worldwide (41, 42). During the study period, the incidence rate of AHB declined steadily—especially among children—largely due to increased HBV vaccination coverage and the availability of antiviral treatments. GBD 2021 data reveal a significant negative correlation between AHB incidence and socioeconomic status, with low-income regions, such as Central Sub-Saharan Africa, bearing the highest burden of disease. Countries like Mauritania exemplified the greatest disparities within lower-middle and low SDI regions. This finding aligns with global HBV vaccination efforts, as coverage for the birth dose of the HBV vaccine was only 41%, with an alarmingly low rate of 16% in African region (43). Low-income regions may be the final battleground for the elimination of HBV; however, high costs associated with testing remain a formidable barrier despite international support. Comprehensive vaccination strategies, prevention of mother-to-child transmission, and the adoption of simplified testing methods (as opposed to gold-standard testing) are proven to be more cost-effective in low-income settings (2, 44, 45).

The continuing rise in new AHC cases is primarily linked to risk factors such as unsafe blood transfusions, intravenous drug use, and male-to-male sexual contact (46). In the United States, intravenous drug use has emerged as the primary risk factor for new HCV infections (47). Without an effective vaccine, the elimination of HCV hinges on the expansion of screening practices and access to antiviral therapies. However, recent studies indicate that, as of 2020, the global diagnosis rate for HCV infections was merely 23% (48). Although direct-acting antiviral (DAA) treatments offer cure rates exceeding 95% (49, 50), the high costs of these medications present significant challenges to achieving global health equity. Egypt stands out as a successful case study among low-middle SDI countries in terms of HCV elimination; as of 2023, its national HCV screening and treatment program attained an 87% diagnosis rate and a 93% cure rate among confirmed cases (51, 52). Furthermore, the National Hepatitis C Elimination Program proposed in the United States aims to extend DAA treatment coverage to incarcerated individuals and uninsured populations, given their high rates of HCV infection (53). To achieve viral hepatitis elimination by 2030, it is imperative to actively promote global health equity at both national and population levels.

This study has several limitations. First, the global estimates of AVH rely on the accuracy and quality of GBD 2021 data; however, there is significant heterogeneity in data collection quality among different countries and regions, particularly in some middle-low-income countries where diagnostic capabilities may severely underestimate the disease burden. Second, due to the low global screening rate for AHD and the lack of high-quality primary data for GBD standardization and modeling, this study regrettably could not provide a comprehensive assessment of the global infection situation of AHD and the associated health disparities. Lastly, there is inadequate differentiation among various viral subtypes; for instance, HEV subtypes exhibit varying epidemiological characteristics, presenting a substantial challenge.

## Conclusion

This study provides an updated assessment of global AVH incidence and epidemiological trends based on GBD 2021 data. From 2000 to 2021, the incidence rates of four major AVH types demonstrated varying degrees of decline, while the number of new cases for AHC and AHE continued to rise. A significant negative correlation exists between AVH incidence and socioeconomic levels. While global health inequalities related to AVH have diminished, certain countries show significant potential for the elimination of viral hepatitis. Promoting health equity at both national and community levels is critical for achieving the goal of eliminating viral hepatitis by 2030.

## Data Availability

The GBD2021 data are available from the online website (https://vizhub.healthdata.org/gbd-results/). Further analyses and visualization results can be obtained from the corresponding author.

## Footnotes

^1^ https://ghdx.healthdata.org/gbd-2021
