# Supplementary material for: Update on the global burden of acute viral hepatitis in 2021: addressing health inequalities
Source: Front Public Health. 2025 May 23;13:1580863. doi: 10.3389/fpubh.2025.1580863 (PMC12141279; doi:10.3389/fpubh.2025.1580863)
Supplement: Supplementary file 1 [file Data_Sheet_1.docx]

**Supplement**

**Update on the global burden of acute viral hepatitis in 2021: addressing health inequalities**

**Content**

**Supplementary methods.**

**Figure S1: The incidence trends of AVH in 21 GBD regions.** (A) ASIR of AVH in each region for the year 2021; (B) APC of ASIR at the regional level from 2000 to 2021.

**Figure S2: The incidence trends of AVH by age and sex.** (A) AHA in 2021; (B) AHB in 2021; (C) AHC in 2021; (D) AHE in 2021; (E) APC of ASIR from 2000 to 2021.

**Table S1.** Annual percentage change in ASIR of AVH at the regional level from 2000 to 2021.

**Table S2.** Annual percentage change in ASIR of AVH at the level of 204 countries and regions from 2000 to 2021.

**Table S3.** Annual percentage change in ASIR of AVH at the level of sociodemographic index from 2000 to 2021.

**Table S4.** Annual percentage change in the incidence of AVH by sex and age from 2000 to 2021.

**Table S5.** Disparity of 204 countries and regions from the frontier ASIR level.

**Table S6.** BAPC model predicts the incidence and ASIR of AVH over the next 15 years.

**Supplementary methods.**

**Data Sources and Study Population**

Estimates of the incidence of acute viral hepatitis are categorized by sex (male and female), age (5-year groups, from <5 years to 95+ years), and SDI (classified into five categories from 0 to 1: low, low-middle, middle, high-middle, and high). Detailed inclusion criteria for each data source can be found at (https://ghdx.healthdata.org/gbd-2021/sources).

**Definition of AVH**

AHA is classified under ICD-10 codes B15, B15.0, and B15.9; AHB under ICD-10 codes B16, B16.0, B16.1, B16.2, B17.0, B19.1, B19.10, B19.11, and P35.3; AHC includes the ICD-10 code B17.1; AHE is categorized by the ICD-10 code B17.2.

**Figures**


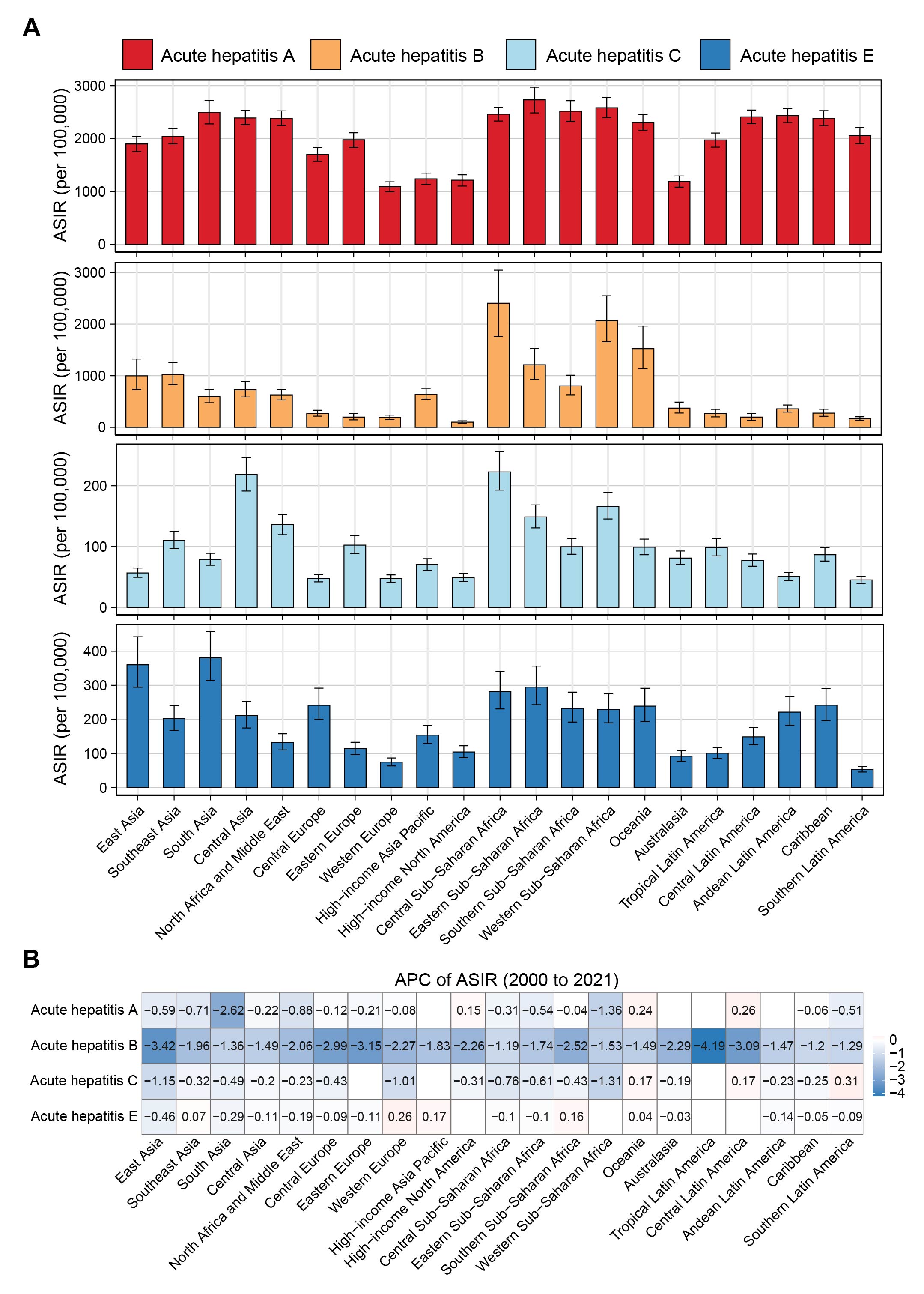


**Figure S1: The incidence trends of AVH in 21 GBD regions.** (A) ASIR of AVH in each region for the year 2021; (B) APC of ASIR at the regional level from 2000 to 2021.


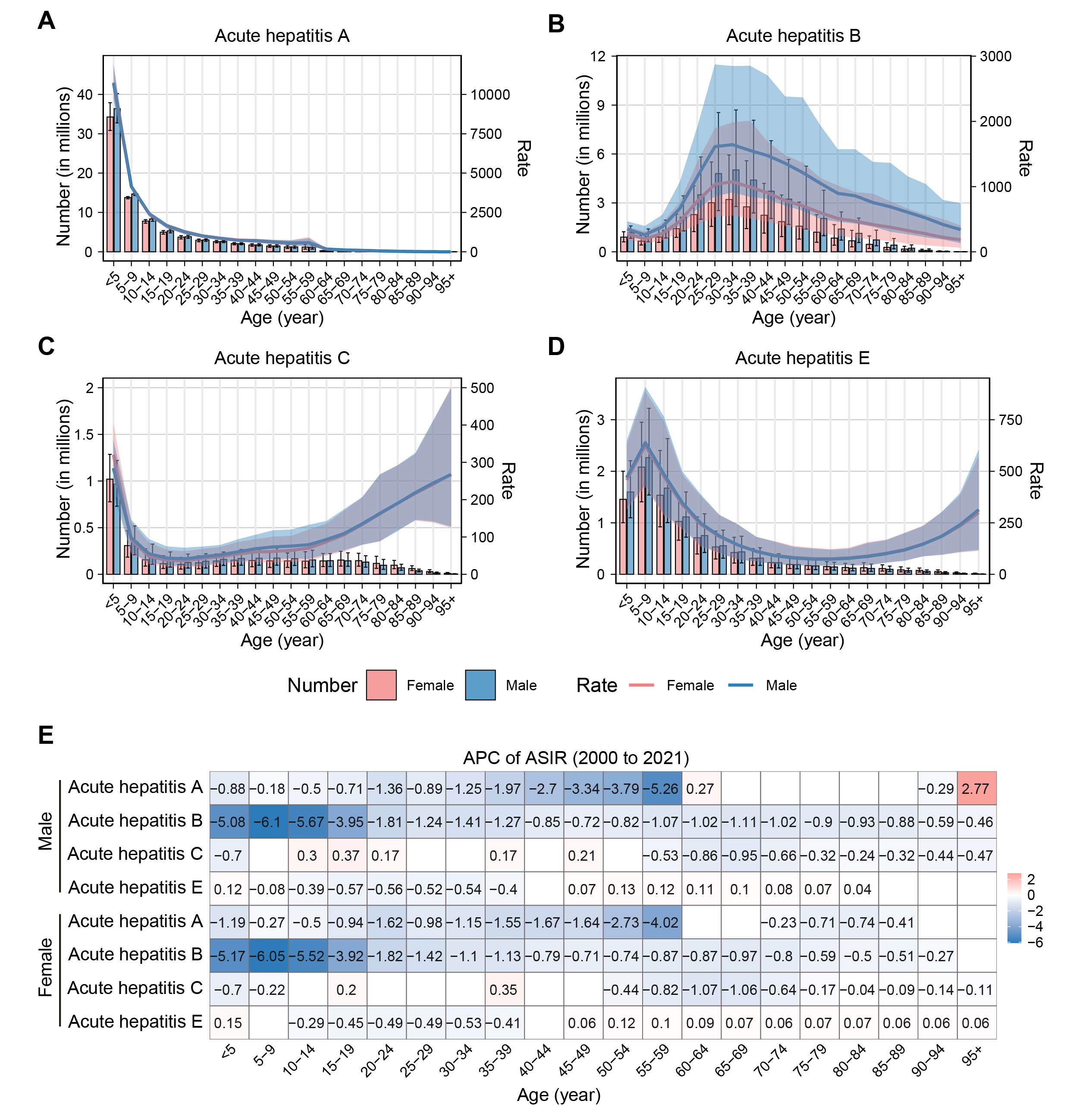


**Figure S2: The incidence trends of AVH by age and sex.** (A) AHA in 2021; (B) AHB in 2021; (C) AHC in 2021; (D) AHE in 2021; (E) APC of ASIR from 2000 to 2021.

**Table S1. Annual percentage change in ASIR of AVH at the regional level from 2000 to 2021**

| location | AHA | AHB | AHC | AHE |
| --- | --- | --- | --- | --- |
| Andean Latin America | 0.02 (-0.00, 0.04) | -1.47 (-1.53, -1.41) | -0.23 (-0.28, -0.18) | -0.14 (-0.16, -0.12) |
| Australasia | -0.07 (-0.24, 0.11) | -2.29 (-2.42, -2.17) | -0.19 (-0.32, -0.06) | -0.03 (-0.04, -0.02) |
| Caribbean | -0.06 (-0.08, -0.05) | -1.20 (-1.27, -1.14) | -0.25 (-0.32, -0.18) | -0.05 (-0.06, -0.04) |
| Central Asia | -0.22 (-0.24, -0.20) | -1.49 (-1.62, -1.37) | -0.20 (-0.28, -0.12) | -0.11 (-0.12, -0.09) |
| Central Europe | -0.12 (-0.16, -0.08) | -2.99 (-3.07, -2.91) | -0.43 (-0.50, -0.36) | -0.09 (-0.11, -0.07) |
| Central Latin America | 0.26 (0.20, 0.33) | -3.09 (-3.15, -3.04) | 0.17 (0.04, 0.31) | 0.03 (-0.02, 0.08) |
| Central Sub-Saharan Africa | -0.31 (-0.35, -0.27) | -1.19 (-1.29, -1.09) | -0.76 (-0.79, -0.72) | -0.10 (-0.10, -0.09) |
| East Asia | -0.59 (-0.64, -0.53) | -3.42 (-3.58, -3.25) | -1.15 (-1.49, -0.81) | -0.46 (-0.53, -0.40) |
| Eastern Europe | -0.21 (-0.34, -0.08) | -3.15 (-3.42, -2.88) | 0.12 (-0.13, 0.37) | -0.11 (-0.15, -0.06) |
| Eastern Sub-Saharan Africa | -0.54 (-0.61, -0.48) | -1.74 (-1.80, -1.68) | -0.61 (-0.68, -0.55) | -0.10 (-0.12, -0.08) |
| High-income Asia Pacific | 0.16 (-0.05, 0.38) | -1.83 (-1.93, -1.74) | -0.51 (-1.54, 0.52) | 0.17 (0.13, 0.22) |
| High-income North America | 0.15 (0.06, 0.25) | -2.26 (-2.42, -2.10) | -0.31 (-0.39, -0.22) | 0.01 (-0.02, 0.04) |
| North Africa and Middle East | -0.88 (-1.01, -0.74) | -2.06 (-2.15, -1.97) | -0.23 (-0.36, -0.09) | -0.19 (-0.26, -0.12) |
| Oceania | 0.24 (0.17, 0.32) | -1.49 (-1.57, -1.40) | 0.17 (0.13, 0.22) | 0.04 (0.02, 0.06) |
| South Asia | -2.62 (-2.82, -2.42) | -1.36 (-1.50, -1.23) | -0.49 (-0.63, -0.34) | -0.29 (-0.34, -0.24) |
| Southeast Asia | -0.71 (-0.77, -0.66) | -1.96 (-2.04, -1.88) | -0.32 (-0.35, -0.29) | 0.07 (0.03, 0.11) |
| Southern Latin America | -0.51 (-0.55, -0.46) | -1.29 (-1.41, -1.18) | 0.31 (0.23, 0.39) | -0.09 (-0.11, -0.07) |
| Southern Sub-Saharan Africa | -0.04 (-0.07, -0.01) | -2.52 (-2.61, -2.42) | -0.43 (-0.48, -0.37) | 0.16 (0.05, 0.27) |
| Tropical Latin America | -0.13 (-0.33, 0.07) | -4.19 (-4.31, -4.07) | -0.12 (-0.61, 0.37) | -0.03 (-0.06, 0.00) |
| Western Europe | -0.08 (-0.11, -0.05) | -2.27 (-2.39, -2.15) | -1.01 (-1.16, -0.85) | 0.26 (0.18, 0.33) |
| Western Sub-Saharan Africa | -1.36 (-1.60, -1.13) | -1.53 (-1.60, -1.45) | -1.31 (-1.37, -1.25) | -0.00 (-0.03, 0.02) |

ASIR, age-standardized incidence rate; AVH, acute viral hepatitis; AHA, acute hepatitis A; AHB, acute hepatitis B; AHC, acute hepatitis C; AHE, acute hepatitis E.

**Table S2. Annual percentage change in ASIR of AVH at the level of 204 countries and regions from 2000 to 2021**

| **location** | **AHA** | **AHB** | **AHC** | **AHE** |
| --- | --- | --- | --- | --- |
| Afghanistan | -4.352 (-4.654, -4.048) | -0.567 (-0.670, -0.464) | -0.791 (-0.872, -0.709) | -0.104 (-0.138, -0.069) |
| Albania | -0.116 (-0.134, -0.098) | -2.497 (-2.672, -2.321) | -1.004 (-1.128, -0.879) | 0.319 (0.221, 0.417) |
| Algeria | -0.133 (-0.146, -0.120) | -1.215 (-1.310, -1.119) | -0.291 (-0.328, -0.255) | -0.173 (-0.194, -0.153) |
| American Samoa | 0.172 (0.110, 0.234) | -2.137 (-2.471, -1.802) | 0.337 (0.291, 0.383) | -0.086 (-0.117, -0.054) |
| Andorra | -0.160 (-0.186, -0.133) | -2.359 (-2.418, -2.300) | 0.023 (-0.098, 0.144) | -0.027 (-0.042, -0.012) |
| Angola | -0.844 (-1.035, -0.652) | -1.119 (-1.223, -1.014) | -0.915 (-0.960, -0.869) | -0.110 (-0.132, -0.087) |
| Antigua and Barbuda | -0.133 (-0.149, -0.117) | -2.445 (-2.633, -2.256) | -0.388 (-0.476, -0.300) | -0.112 (-0.137, -0.088) |
| Argentina | -0.706 (-0.741, -0.672) | -1.304 (-1.432, -1.177) | 0.530 (0.450, 0.611) | 0.028 (-0.033, 0.088) |
| Armenia | -0.218 (-0.258, -0.178) | -1.728 (-1.830, -1.626) | 0.279 (0.081, 0.477) | -0.115 (-0.142, -0.088) |
| Australia | -0.098 (-0.293, 0.098) | -2.251 (-2.398, -2.102) | -0.293 (-0.434, -0.151) | -0.027 (-0.038, -0.016) |
| Austria | -0.135 (-0.160, -0.109) | -2.167 (-2.235, -2.099) | -0.795 (-0.878, -0.712) | -0.041 (-0.063, -0.018) |
| Azerbaijan | -0.318 (-0.351, -0.284) | -1.127 (-1.190, -1.065) | -0.724 (-0.777, -0.670) | -0.220 (-0.244, -0.197) |
| Bahamas | -0.085 (-0.097, -0.073) | -2.123 (-2.277, -1.968) | -0.345 (-0.419, -0.270) | -0.045 (-0.057, -0.034) |
| Bahrain | -0.075 (-0.087, -0.062) | -1.938 (-2.118, -1.756) | -0.783 (-0.835, -0.731) | -0.097 (-0.119, -0.076) |
| Bangladesh | -0.674 (-0.873, -0.474) | -1.320 (-1.498, -1.142) | -0.534 (-0.882, -0.186) | -0.779 (-0.927, -0.630) |
| Barbados | -0.129 (-0.142, -0.116) | -2.137 (-2.192, -2.082) | -0.302 (-0.347, -0.258) | -0.071 (-0.083, -0.060) |
| Belarus | -0.324 (-0.390, -0.259) | -3.370 (-3.547, -3.193) | -0.046 (-0.293, 0.202) | -0.068 (-0.094, -0.041) |
| Belgium | -0.104 (-0.128, -0.081) | -2.289 (-2.374, -2.205) | 0.130 (0.040, 0.221) | 0.030 (-0.039, 0.100) |
| Belize | -0.068 (-0.077, -0.058) | -2.092 (-2.214, -1.970) | -0.455 (-0.513, -0.397) | -0.101 (-0.116, -0.085) |
| Benin | -0.653 (-0.677, -0.629) | -1.137 (-1.301, -0.973) | -0.776 (-0.785, -0.767) | -0.065 (-0.089, -0.041) |
| Bermuda | -0.094 (-0.107, -0.081) | -1.988 (-2.104, -1.873) | -0.740 (-0.821, -0.658) | -0.043 (-0.055, -0.031) |
| Bhutan | -0.127 (-0.269, 0.014) | -2.574 (-2.680, -2.466) | 0.837 (0.664, 1.010) | 0.815 (0.368, 1.263) |
| Bolivia (Plurinational State of) | 0.035 (-0.082, 0.152) | -0.998 (-1.089, -0.906) | -0.310 (-0.327, -0.292) | -0.130 (-0.153, -0.106) |
| Bosnia and Herzegovina | -0.246 (-0.284, -0.207) | -1.568 (-1.638, -1.498) | 0.281 (0.135, 0.428) | -0.143 (-0.181, -0.105) |
| Botswana | -0.075 (-0.092, -0.059) | -2.717 (-2.849, -2.585) | -0.465 (-0.515, -0.415) | -0.114 (-0.124, -0.103) |
| Brazil | -0.115 (-0.324, 0.095) | -4.250 (-4.371, -4.129) | -0.115 (-0.616, 0.389) | -0.024 (-0.056, 0.007) |
| Brunei Darussalam | -0.031 (-0.046, -0.016) | -3.587 (-3.748, -3.427) | -0.028 (-0.087, 0.031) | -0.002 (-0.015, 0.012) |
| Bulgaria | 0.021 (-0.002, 0.044) | -3.126 (-3.330, -2.921) | -0.241 (-0.464, -0.018) | -0.089 (-0.107, -0.072) |
| Burkina Faso | -0.642 (-0.723, -0.561) | -1.641 (-1.808, -1.474) | -0.894 (-0.964, -0.824) | -0.035 (-0.055, -0.015) |
| Burundi | -0.207 (-0.235, -0.178) | -1.781 (-1.887, -1.676) | -0.641 (-0.701, -0.581) | -0.058 (-0.075, -0.041) |
| C?te d'Ivoire | -0.518 (-0.535, -0.500) | -1.669 (-1.758, -1.580) | -0.663 (-0.703, -0.624) | -0.052 (-0.066, -0.037) |
| Cabo Verde | -0.290 (-0.327, -0.254) | -1.950 (-2.093, -1.807) | -0.791 (-0.868, -0.714) | -0.148 (-0.167, -0.129) |
| Cambodia | -3.120 (-3.578, -2.661) | -2.272 (-2.467, -2.076) | -0.670 (-0.797, -0.542) | -0.096 (-0.110, -0.082) |
| Cameroon | -0.189 (-0.202, -0.175) | -1.233 (-1.317, -1.149) | -1.042 (-1.095, -0.988) | -0.056 (-0.078, -0.034) |
| Canada | 1.248 (1.055, 1.442) | -1.623 (-1.702, -1.544) | 0.065 (-0.018, 0.148) | -0.018 (-0.039, 0.002) |
| Central African Republic | -0.434 (-0.480, -0.388) | -0.831 (-0.921, -0.740) | -0.279 (-0.399, -0.158) | -0.030 (-0.042, -0.019) |
| Chad | -0.957 (-1.095, -0.819) | -0.700 (-0.792, -0.608) | -0.580 (-0.620, -0.540) | -0.047 (-0.071, -0.022) |
| Chile | 0.081 (0.013, 0.150) | -1.233 (-1.344, -1.122) | 0.003 (-0.130, 0.137) | -0.111 (-0.136, -0.085) |
| China | -0.613 (-0.670, -0.554) | -3.450 (-3.628, -3.272) | -1.118 (-1.472, -0.763) | -0.490 (-0.550, -0.429) |
| Colombia | -0.090 (-0.176, -0.004) | -3.247 (-3.411, -3.083) | -1.039 (-1.318, -0.759) | -0.135 (-0.147, -0.123) |
| Comoros | -0.177 (-0.205, -0.149) | -1.750 (-1.849, -1.651) | -0.510 (-0.560, -0.461) | -0.077 (-0.094, -0.060) |
| Congo | -0.434 (-0.457, -0.412) | -1.590 (-1.649, -1.531) | -0.605 (-0.670, -0.539) | -0.109 (-0.123, -0.094) |
| Cook Islands | 0.156 (0.102, 0.210) | -3.211 (-3.525, -2.896) | -0.331 (-0.358, -0.305) | -0.065 (-0.084, -0.045) |
| Costa Rica | 0.042 (0.027, 0.056) | -2.297 (-2.506, -2.087) | -0.312 (-0.346, -0.278) | -0.076 (-0.098, -0.055) |
| Croatia | 0.084 (-0.221, 0.390) | -2.245 (-2.364, -2.125) | -0.598 (-0.688, -0.508) | -0.095 (-0.116, -0.074) |
| Cuba | -0.091 (-0.121, -0.061) | -3.397 (-3.577, -3.216) | -0.433 (-0.485, -0.380) | 0.269 (0.179, 0.358) |
| Cyprus | 0.090 (0.051, 0.129) | -3.013 (-3.088, -2.938) | -0.055 (-0.119, 0.010) | -0.021 (-0.038, -0.005) |
| Czechia | 0.257 (0.074, 0.439) | -2.309 (-2.463, -2.155) | 0.044 (-0.096, 0.184) | -0.071 (-0.099, -0.044) |
| Democratic People's Republic of Korea | -0.473 (-0.505, -0.440) | -2.108 (-2.222, -1.993) | -0.198 (-0.285, -0.111) | -0.175 (-0.195, -0.156) |
| Democratic Republic of the Congo | -0.119 (-0.134, -0.105) | -1.216 (-1.345, -1.087) | -0.720 (-0.774, -0.665) | -0.082 (-0.092, -0.073) |
| Denmark | -0.003 (-0.043, 0.036) | -0.051 (-0.105, 0.003) | -0.475 (-0.561, -0.389) | -0.014 (-0.028, -0.000) |
| Djibouti | -0.226 (-0.236, -0.216) | -1.082 (-1.181, -0.983) | -0.494 (-0.526, -0.462) | -0.068 (-0.085, -0.051) |
| Dominica | -0.121 (-0.134, -0.109) | -1.918 (-2.087, -1.749) | -0.594 (-0.669, -0.518) | -0.114 (-0.121, -0.106) |
| Dominican Republic | -0.251 (-0.308, -0.195) | -1.671 (-1.788, -1.553) | -0.118 (-0.210, -0.025) | -0.136 (-0.144, -0.128) |
| Ecuador | 0.129 (0.112, 0.146) | -1.236 (-1.350, -1.122) | -0.187 (-0.260, -0.114) | -0.124 (-0.146, -0.101) |
| Egypt | -1.796 (-1.999, -1.592) | -2.567 (-2.898, -2.234) | -0.328 (-0.633, -0.021) | -0.211 (-0.237, -0.185) |
| El Salvador | -0.039 (-0.048, -0.030) | -2.758 (-2.864, -2.652) | -0.331 (-0.368, -0.294) | -0.087 (-0.112, -0.062) |
| Equatorial Guinea | -0.242 (-0.254, -0.230) | -0.664 (-0.805, -0.523) | -1.272 (-1.382, -1.162) | -0.244 (-0.273, -0.216) |
| Eritrea | -0.424 (-0.461, -0.387) | -1.990 (-2.127, -1.853) | -0.262 (-0.282, -0.242) | -0.086 (-0.100, -0.073) |
| Estonia | -0.192 (-0.220, -0.163) | -2.722 (-2.867, -2.576) | -0.379 (-0.463, -0.294) | 0.007 (-0.018, 0.033) |
| Eswatini | -0.380 (-0.414, -0.345) | -2.507 (-2.687, -2.326) | -0.193 (-0.267, -0.119) | -0.029 (-0.050, -0.009) |
| Ethiopia | -0.882 (-1.044, -0.720) | -1.481 (-1.540, -1.422) | -0.827 (-0.997, -0.656) | -0.073 (-0.096, -0.051) |
| Fiji | 0.075 (0.024, 0.126) | -2.850 (-3.073, -2.627) | 0.133 (0.113, 0.152) | -0.129 (-0.156, -0.103) |
| Finland | -0.073 (-0.240, 0.094) | -0.251 (-0.283, -0.219) | -0.325 (-0.365, -0.284) | -0.034 (-0.048, -0.019) |
| France | -0.677 (-0.831, -0.522) | -1.534 (-1.627, -1.441) | 0.617 (0.359, 0.876) | 0.473 (0.364, 0.582) |
| Gabon | -0.200 (-0.216, -0.184) | -1.590 (-1.730, -1.450) | -1.366 (-1.515, -1.217) | 0.033 (0.004, 0.062) |
| Gambia | -0.098 (-0.111, -0.086) | -2.308 (-2.518, -2.098) | -0.236 (-0.328, -0.144) | -0.037 (-0.058, -0.015) |
| Georgia | -0.226 (-0.245, -0.207) | -1.532 (-1.633, -1.431) | -0.605 (-0.946, -0.263) | -0.104 (-0.120, -0.088) |
| Germany | 0.469 (0.330, 0.608) | -2.067 (-2.192, -1.942) | -0.187 (-0.454, 0.080) | -0.025 (-0.037, -0.012) |
| Ghana | -1.287 (-1.368, -1.206) | -2.118 (-2.292, -1.944) | -0.934 (-1.026, -0.842) | -0.065 (-0.086, -0.044) |
| Greece | -0.419 (-0.458, -0.381) | -2.168 (-2.257, -2.079) | 0.196 (0.039, 0.354) | 0.198 (0.101, 0.296) |
| Greenland | -0.139 (-0.157, -0.121) | -1.177 (-1.294, -1.059) | -0.232 (-0.278, -0.186) | -0.053 (-0.075, -0.030) |
| Grenada | -0.155 (-0.173, -0.137) | -2.213 (-2.369, -2.057) | -0.689 (-0.769, -0.609) | -0.095 (-0.106, -0.084) |
| Guam | 0.217 (0.164, 0.271) | -1.892 (-2.206, -1.576) | 0.303 (0.203, 0.403) | -0.045 (-0.060, -0.031) |
| Guatemala | 0.043 (0.028, 0.058) | -2.031 (-2.142, -1.919) | -0.649 (-0.740, -0.557) | -0.106 (-0.119, -0.094) |
| Guinea | -1.037 (-1.132, -0.942) | -0.928 (-1.066, -0.790) | -0.687 (-0.713, -0.661) | -0.076 (-0.106, -0.046) |
| Guinea-Bissau | -1.316 (-1.457, -1.175) | -1.555 (-1.679, -1.431) | 0.277 (-0.017, 0.572) | -0.046 (-0.071, -0.021) |
| Guyana | -0.191 (-0.204, -0.178) | -2.115 (-2.365, -1.865) | -0.375 (-0.422, -0.328) | -0.119 (-0.133, -0.106) |
| Haiti | -0.033 (-0.041, -0.025) | -0.841 (-0.948, -0.734) | -0.763 (-0.835, -0.691) | -0.092 (-0.102, -0.082) |
| Honduras | -0.146 (-0.150, -0.141) | -2.369 (-2.578, -2.159) | -0.515 (-0.525, -0.504) | -0.133 (-0.144, -0.122) |
| Hungary | 0.153 (-0.006, 0.313) | -0.004 (-0.032, 0.024) | -1.282 (-1.408, -1.155) | -0.082 (-0.104, -0.059) |
| Iceland | 0.302 (0.192, 0.413) | -0.341 (-0.405, -0.278) | -0.416 (-0.447, -0.386) | 0.004 (-0.015, 0.024) |
| India | -3.085 (-3.380, -2.790) | -1.433 (-1.593, -1.274) | -0.859 (-1.160, -0.557) | -0.190 (-0.230, -0.150) |
| Indonesia | -1.106 (-1.224, -0.987) | -2.101 (-2.153, -2.049) | -0.348 (-0.388, -0.307) | 0.141 (0.058, 0.224) |
| Iran (Islamic Republic of) | 0.120 (-0.170, 0.411) | -3.479 (-3.546, -3.412) | -0.070 (-1.151, 1.024) | 0.215 (-0.085, 0.516) |
| Iraq | -0.334 (-0.368, -0.301) | -1.081 (-1.196, -0.966) | 0.671 (0.480, 0.863) | -0.120 (-0.165, -0.076) |
| Ireland | -0.250 (-0.299, -0.200) | -1.686 (-1.868, -1.504) | -0.225 (-0.275, -0.175) | -0.025 (-0.038, -0.011) |
| Israel | -0.742 (-0.893, -0.590) | -2.612 (-2.742, -2.481) | -0.698 (-0.762, -0.635) | 0.042 (0.009, 0.075) |
| Italy | 0.137 (-0.032, 0.307) | -4.569 (-4.675, -4.464) | -2.679 (-3.103, -2.254) | 0.706 (0.408, 1.004) |
| Jamaica | 0.015 (-0.081, 0.111) | -2.588 (-2.741, -2.436) | -0.200 (-0.254, -0.146) | -0.087 (-0.097, -0.077) |
| Japan | 0.892 (0.651, 1.133) | -1.400 (-1.531, -1.270) | -0.537 (-1.878, 0.823) | 0.004 (-0.013, 0.021) |
| Jordan | -0.012 (-0.081, 0.057) | -2.716 (-2.814, -2.619) | -0.169 (-0.701, 0.366) | -0.161 (-0.209, -0.112) |
| Kazakhstan | -0.114 (-0.185, -0.044) | -1.512 (-1.629, -1.394) | 0.165 (0.008, 0.323) | -0.110 (-0.135, -0.085) |
| Kenya | -0.426 (-0.488, -0.364) | -3.015 (-3.273, -2.757) | -0.141 (-0.155, -0.126) | -0.069 (-0.095, -0.042) |
| Kiribati | -1.125 (-1.310, -0.939) | -1.538 (-1.822, -1.254) | 0.479 (0.425, 0.533) | -0.060 (-0.078, -0.042) |
| Kuwait | -0.053 (-0.060, -0.046) | -2.668 (-2.855, -2.481) | -0.258 (-0.368, -0.147) | -0.139 (-0.174, -0.104) |
| Kyrgyzstan | -0.247 (-0.273, -0.220) | -1.753 (-1.854, -1.652) | -0.337 (-0.549, -0.124) | -0.103 (-0.115, -0.090) |
| Lao People's Democratic Republic | -1.362 (-1.576, -1.147) | -1.686 (-1.749, -1.623) | -0.920 (-1.052, -0.787) | -0.023 (-0.035, -0.012) |
| Latvia | -0.170 (-0.197, -0.143) | -2.799 (-2.956, -2.642) | -0.025 (-0.142, 0.092) | -0.018 (-0.060, 0.023) |
| Lebanon | -0.073 (-0.119, -0.028) | -1.317 (-1.387, -1.247) | 0.303 (-0.139, 0.746) | -0.172 (-0.281, -0.064) |
| Lesotho | -0.275 (-0.336, -0.214) | -1.968 (-2.071, -1.865) | 0.180 (0.165, 0.194) | -0.004 (-0.026, 0.019) |
| Liberia | -0.844 (-0.903, -0.785) | -1.142 (-1.258, -1.026) | -0.816 (-0.861, -0.772) | -0.036 (-0.063, -0.009) |
| Libya | -0.125 (-0.153, -0.098) | -1.428 (-1.567, -1.288) | 1.416 (0.804, 2.031) | -0.162 (-0.189, -0.134) |
| Lithuania | -0.129 (-0.156, -0.102) | -2.931 (-3.041, -2.821) | -0.188 (-0.284, -0.092) | 0.007 (-0.017, 0.031) |
| Luxembourg | -0.165 (-0.188, -0.141) | -2.309 (-2.464, -2.153) | -1.008 (-1.067, -0.949) | -0.036 (-0.056, -0.016) |
| Macedonia | -0.322 (-0.386, -0.259) | -1.536 (-1.584, -1.489) | -0.143 (-0.186, -0.099) | -0.065 (-0.081, -0.050) |
| Madagascar | 0.131 (0.047, 0.215) | -1.583 (-1.757, -1.409) | 0.391 (0.267, 0.514) | -0.035 (-0.066, -0.005) |
| Malawi | -0.736 (-0.787, -0.684) | -2.090 (-2.187, -1.993) | -0.847 (-0.897, -0.797) | -0.072 (-0.086, -0.059) |
| Malaysia | -0.134 (-0.154, -0.115) | -2.455 (-2.534, -2.376) | -0.875 (-0.969, -0.780) | -0.009 (-0.019, 0.000) |
| Maldives | -0.377 (-0.404, -0.350) | -2.394 (-2.467, -2.321) | -1.089 (-1.225, -0.953) | -0.068 (-0.083, -0.052) |
| Mali | -0.080 (-0.092, -0.067) | -1.302 (-1.443, -1.161) | -1.023 (-1.065, -0.982) | -0.040 (-0.062, -0.017) |
| Malta | -0.121 (-0.151, -0.092) | -2.444 (-2.625, -2.262) | -0.582 (-0.624, -0.540) | -0.049 (-0.068, -0.030) |
| Marshall Islands | -0.176 (-0.209, -0.143) | -2.395 (-2.690, -2.098) | 0.234 (0.204, 0.265) | -0.124 (-0.135, -0.113) |
| Mauritania | -1.450 (-1.597, -1.302) | -1.401 (-1.538, -1.263) | -0.922 (-0.961, -0.884) | -0.055 (-0.077, -0.033) |
| Mauritius | -0.242 (-0.256, -0.228) | -2.400 (-2.512, -2.288) | -0.740 (-0.769, -0.712) | -0.006 (-0.020, 0.007) |
| Mexico | 0.554 (0.432, 0.677) | -3.719 (-3.877, -3.560) | 1.155 (0.789, 1.522) | 0.124 (0.024, 0.224) |
| Micronesia (Federated States of) | 0.099 (-0.086, 0.284) | -2.405 (-2.672, -2.137) | 0.264 (0.208, 0.320) | -0.090 (-0.102, -0.077) |
| Monaco | -0.155 (-0.185, -0.125) | -3.060 (-3.245, -2.874) | 0.389 (0.296, 0.482) | -0.022 (-0.043, -0.001) |
| Mongolia | -0.281 (-0.303, -0.259) | -2.032 (-2.104, -1.961) | 0.033 (-0.234, 0.301) | 0.311 (0.216, 0.405) |
| Montenegro | -0.199 (-0.219, -0.178) | -1.904 (-2.022, -1.787) | -0.380 (-0.410, -0.349) | -0.119 (-0.138, -0.100) |
| Morocco | -0.292 (-0.314, -0.271) | -1.548 (-1.661, -1.435) | 0.961 (0.583, 1.340) | -0.227 (-0.261, -0.193) |
| Mozambique | -0.318 (-0.345, -0.292) | -1.772 (-1.912, -1.632) | -0.198 (-0.232, -0.163) | -0.058 (-0.072, -0.043) |
| Myanmar | -0.689 (-0.747, -0.632) | -1.342 (-1.419, -1.266) | -0.469 (-0.542, -0.396) | -0.040 (-0.058, -0.022) |
| Namibia | -0.125 (-0.148, -0.102) | -1.785 (-1.921, -1.650) | -0.370 (-0.482, -0.259) | -0.039 (-0.045, -0.033) |
| Nauru | -0.061 (-0.098, -0.024) | -3.124 (-3.447, -2.801) | 0.217 (0.180, 0.254) | -0.134 (-0.160, -0.108) |
| Nepal | -1.371 (-1.532, -1.209) | -0.963 (-1.056, -0.870) | 0.669 (0.343, 0.996) | -0.141 (-0.159, -0.122) |
| Netherlands | 0.227 (0.094, 0.359) | -1.013 (-1.130, -0.896) | 0.029 (-0.409, 0.470) | 0.478 (-0.028, 0.988) |
| New Zealand | 0.071 (-0.012, 0.153) | -2.586 (-2.680, -2.490) | 0.398 (0.290, 0.506) | -0.014 (-0.027, -0.001) |
| Nicaragua | -0.002 (-0.036, 0.031) | -2.519 (-2.681, -2.356) | -0.033 (-0.075, 0.009) | -0.193 (-0.213, -0.172) |
| Niger | -0.820 (-0.873, -0.768) | -1.078 (-1.186, -0.970) | -0.707 (-0.748, -0.666) | -0.028 (-0.054, -0.002) |
| Nigeria | -1.865 (-2.275, -1.453) | -1.572 (-1.648, -1.495) | -1.853 (-1.963, -1.743) | 0.021 (-0.008, 0.051) |
| Niue | 0.130 (0.070, 0.190) | -3.685 (-4.111, -3.256) | -0.175 (-0.238, -0.113) | -0.050 (-0.072, -0.027) |
| Northern Mariana Islands | 0.250 (0.205, 0.294) | -1.728 (-1.948, -1.507) | 0.102 (0.037, 0.167) | -0.001 (-0.030, 0.027) |
| Norway | -0.054 (-0.076, -0.032) | -3.066 (-3.235, -2.897) | 0.237 (0.051, 0.423) | -0.040 (-0.063, -0.016) |
| Oman | -1.025 (-1.101, -0.949) | -2.866 (-3.106, -2.625) | -0.689 (-0.741, -0.636) | -0.205 (-0.240, -0.171) |
| Pakistan | -1.642 (-1.722, -1.561) | -1.274 (-1.404, -1.143) | -0.287 (-0.760, 0.188) | -0.028 (-0.045, -0.012) |
| Palau | -0.065 (-0.142, 0.013) | -2.736 (-2.984, -2.488) | 0.214 (0.197, 0.232) | -0.021 (-0.037, -0.005) |
| Palestine | 0.090 (0.080, 0.101) | -2.315 (-2.445, -2.185) | -0.525 (-0.582, -0.468) | -0.128 (-0.153, -0.103) |
| Panama | -0.049 (-0.070, -0.027) | -2.360 (-2.583, -2.137) | -0.361 (-0.437, -0.284) | -0.172 (-0.191, -0.153) |
| Papua New Guinea | 0.243 (0.157, 0.330) | -1.569 (-1.681, -1.457) | -0.004 (-0.068, 0.059) | 0.011 (-0.012, 0.034) |
| Paraguay | -0.491 (-0.535, -0.448) | -2.006 (-2.187, -1.825) | -0.111 (-0.145, -0.076) | -0.098 (-0.134, -0.062) |
| Peru | -0.035 (-0.041, -0.029) | -1.606 (-1.688, -1.524) | -0.212 (-0.278, -0.145) | -0.148 (-0.167, -0.130) |
| Philippines | -0.172 (-0.200, -0.144) | -2.193 (-2.337, -2.049) | -0.330 (-0.389, -0.270) | -0.035 (-0.051, -0.020) |
| Poland | 0.299 (0.229, 0.368) | -5.192 (-5.316, -5.068) | -0.075 (-0.182, 0.031) | -0.093 (-0.108, -0.077) |
| Portugal | -0.512 (-0.602, -0.422) | -2.620 (-2.752, -2.488) | 0.348 (0.241, 0.455) | -0.038 (-0.120, 0.044) |
| Puerto Rico | -0.082 (-0.087, -0.077) | -2.847 (-2.897, -2.797) | -0.938 (-1.038, -0.837) | -0.057 (-0.074, -0.040) |
| Qatar | -0.048 (-0.055, -0.041) | -2.222 (-2.358, -2.086) | -0.941 (-1.023, -0.859) | -0.072 (-0.100, -0.045) |
| Republic of Korea | -0.546 (-0.836, -0.255) | -2.675 (-2.746, -2.604) | -0.343 (-0.832, 0.148) | 0.479 (0.324, 0.635) |
| Republic of Moldova | -0.314 (-0.337, -0.290) | -2.821 (-2.951, -2.691) | -0.550 (-0.822, -0.277) | -0.067 (-0.081, -0.054) |
| Romania | -0.568 (-0.667, -0.470) | -2.667 (-2.905, -2.429) | -0.719 (-0.940, -0.498) | -0.185 (-0.220, -0.150) |
| Russian Federation | -0.418 (-0.575, -0.261) | -3.585 (-3.885, -3.284) | 0.110 (-0.090, 0.309) | 0.061 (0.005, 0.117) |
| Rwanda | -0.710 (-0.905, -0.515) | -2.413 (-2.512, -2.314) | -1.175 (-1.299, -1.050) | -0.074 (-0.096, -0.051) |
| Saint Kitts and Nevis | -0.139 (-0.150, -0.127) | -2.672 (-2.846, -2.497) | -0.755 (-0.908, -0.602) | -0.089 (-0.099, -0.079) |
| Saint Lucia | -0.183 (-0.199, -0.168) | -2.098 (-2.198, -1.999) | -0.497 (-0.647, -0.347) | -0.112 (-0.127, -0.097) |
| Saint Vincent and the Grenadines | -0.147 (-0.157, -0.136) | -2.318 (-2.456, -2.181) | -0.349 (-0.436, -0.263) | -0.101 (-0.108, -0.093) |
| Samoa | 0.176 (0.112, 0.240) | -1.498 (-1.735, -1.260) | 0.724 (0.655, 0.793) | -0.079 (-0.100, -0.059) |
| San Marino | -0.151 (-0.173, -0.128) | -1.902 (-2.107, -1.696) | 0.131 (0.076, 0.186) | -0.007 (-0.012, -0.002) |
| Sao,me and Principe | -0.560 (-0.608, -0.513) | -1.909 (-2.007, -1.810) | -0.925 (-0.953, -0.897) | -0.160 (-0.196, -0.123) |
| Saudi Arabia | -0.420 (-0.465, -0.375) | -2.530 (-2.636, -2.424) | -0.574 (-0.670, -0.479) | -0.199 (-0.222, -0.176) |
| Senegal | -0.160 (-0.172, -0.148) | -1.774 (-1.845, -1.702) | -0.018 (-0.046, 0.009) | -0.044 (-0.064, -0.024) |
| Serbia | -0.213 (-0.244, -0.182) | -1.805 (-1.913, -1.697) | -0.484 (-0.520, -0.448) | -0.135 (-0.167, -0.103) |
| Seychelles | -0.444 (-0.471, -0.417) | -2.255 (-2.488, -2.021) | -0.219 (-0.263, -0.176) | -0.031 (-0.043, -0.018) |
| Sierra Leone | -1.275 (-1.390, -1.159) | -1.611 (-1.651, -1.571) | -1.009 (-1.059, -0.960) | -0.025 (-0.060, 0.009) |
| Singapore | -0.067 (-0.082, -0.052) | -2.669 (-2.793, -2.545) | 0.028 (-0.038, 0.094) | -0.028 (-0.044, -0.012) |
| Slovakia | -0.074 (-0.087, -0.061) | -2.459 (-2.561, -2.356) | -0.103 (-0.220, 0.013) | -0.080 (-0.104, -0.055) |
| Slovenia | -0.075 (-0.083, -0.067) | -2.037 (-2.152, -1.921) | -0.462 (-0.486, -0.437) | -0.083 (-0.116, -0.051) |
| Solomon Islands | -0.100 (-0.142, -0.058) | -2.818 (-3.043, -2.593) | 0.439 (0.374, 0.505) | -0.071 (-0.089, -0.052) |
| Somalia | -0.835 (-0.875, -0.793) | -0.380 (-0.431, -0.329) | -0.352 (-0.377, -0.327) | -0.061 (-0.079, -0.043) |
| South Africa | 0.016 (0.011, 0.021) | -3.427 (-3.584, -3.269) | -0.692 (-0.760, -0.624) | 0.250 (0.095, 0.406) |
| South Sudan | 0.069 (0.045, 0.093) | -0.580 (-0.701, -0.458) | -0.300 (-0.328, -0.272) | -0.032 (-0.058, -0.007) |
| Spain | 0.541 (0.349, 0.734) | -2.780 (-2.979, -2.582) | -0.774 (-0.825, -0.724) | 0.989 (0.743, 1.235) |
| Sri Lanka | -0.126 (-0.180, -0.072) | -1.719 (-1.765, -1.673) | -0.218 (-0.338, -0.097) | -0.095 (-0.115, -0.075) |
| Sudan | -1.612 (-1.752, -1.472) | -1.743 (-1.783, -1.703) | -0.744 (-0.780, -0.708) | -0.093 (-0.134, -0.052) |
| Suriname | -0.199 (-0.207, -0.190) | -2.049 (-2.240, -1.856) | -0.431 (-0.508, -0.355) | -0.100 (-0.113, -0.087) |
| Sweden | -0.770 (-1.062, -0.477) | -0.374 (-0.617, -0.131) | 0.173 (0.090, 0.256) | -0.007 (-0.015, 0.000) |
| Switzerland | -0.113 (-0.120, -0.106) | -0.545 (-0.658, -0.432) | -0.250 (-0.297, -0.202) | 0.032 (-0.003, 0.067) |
| Syrian Arab Republic | -0.060 (-0.087, -0.034) | -1.596 (-1.688, -1.504) | -0.085 (-0.130, -0.040) | -0.188 (-0.212, -0.165) |
| Taiwan | 0.612 (0.434, 0.789) | -4.068 (-4.358, -3.777) | -1.302 (-1.478, -1.126) | 0.405 (0.291, 0.518) |
| Tajikistan | -0.097 (-0.111, -0.083) | -1.597 (-1.710, -1.484) | -0.346 (-0.355, -0.337) | -0.108 (-0.129, -0.088) |
| Thailand | 1.099 (0.581, 1.619) | -2.238 (-2.405, -2.071) | -0.435 (-0.695, -0.174) | 0.151 (0.061, 0.242) |
| Timor-Leste | -0.267 (-0.283, -0.250) | -1.323 (-1.409, -1.236) | -0.474 (-0.586, -0.361) | 0.002 (-0.017, 0.021) |
| Togo | -0.161 (-0.201, -0.121) | -1.528 (-1.650, -1.406) | -0.809 (-0.844, -0.774) | -0.053 (-0.081, -0.025) |
| Tokelau | 0.060 (-0.002, 0.122) | -2.641 (-2.955, -2.326) | -0.124 (-0.167, -0.082) | -0.130 (-0.161, -0.100) |
| Tonga | -0.803 (-0.895, -0.711) | -3.158 (-3.471, -2.844) | 0.222 (0.198, 0.245) | -0.082 (-0.106, -0.057) |
| Trinidad and,bago | -0.251 (-0.289, -0.213) | -1.681 (-1.797, -1.564) | -0.467 (-0.563, -0.370) | -0.122 (-0.145, -0.098) |
| Tunisia | -0.054 (-0.061, -0.046) | -2.343 (-2.514, -2.173) | 0.190 (-0.059, 0.438) | -0.052 (-0.123, 0.019) |
| Türkiye | -0.338 (-0.735, 0.060) | -1.976 (-2.079, -1.873) | -0.089 (-0.654, 0.479) | -0.329 (-0.547, -0.110) |
| Turkmenistan | -0.338 (-0.360, -0.315) | -1.632 (-1.752, -1.512) | 0.217 (0.112, 0.321) | -0.165 (-0.184, -0.145) |
| Tuvalu | 0.049 (-0.018, 0.116) | -1.764 (-1.998, -1.528) | 0.371 (0.352, 0.390) | -0.077 (-0.092, -0.062) |
| Uganda | -0.567 (-0.643, -0.491) | -1.829 (-1.873, -1.785) | -0.976 (-1.041, -0.910) | -0.089 (-0.116, -0.062) |
| Ukraine | 0.463 (0.349, 0.577) | -1.459 (-1.772, -1.144) | 0.509 (0.103, 0.916) | 0.021 (-0.013, 0.055) |
| United Arab Emirates | 0.089 (0.050, 0.129) | -1.962 (-2.146, -1.777) | -0.141 (-0.214, -0.067) | -0.094 (-0.123, -0.065) |
| United Kingdom | 0.021 (-0.053, 0.096) | -0.254 (-0.452, -0.055) | -1.044 (-2.119, 0.043) | -0.002 (-0.014, 0.010) |
| United Republic of Tanzania | -0.275 (-0.297, -0.252) | -2.002 (-2.141, -1.863) | -0.778 (-0.904, -0.652) | -0.036 (-0.075, 0.002) |
| United States of America | 0.086 (-0.009, 0.182) | -2.640 (-2.884, -2.395) | -0.331 (-0.427, -0.235) | 0.018 (-0.015, 0.050) |
| United States Virgin Islands | -0.108 (-0.127, -0.089) | -2.345 (-2.442, -2.248) | -0.489 (-0.564, -0.415) | -0.067 (-0.078, -0.056) |
| Uruguay | -0.537 (-0.687, -0.387) | -1.825 (-1.945, -1.705) | -0.309 (-0.390, -0.228) | -0.032 (-0.081, 0.016) |
| Uzbekistan | -0.205 (-0.231, -0.180) | -1.766 (-1.960, -1.573) | -0.142 (-0.257, -0.028) | -0.127 (-0.134, -0.121) |
| Vanuatu | 0.068 (0.026, 0.110) | -1.870 (-2.223, -1.517) | 0.259 (0.233, 0.285) | -0.058 (-0.076, -0.039) |
| Venezuela (Bolivarian Republic of) | 0.091 (0.060, 0.123) | -2.152 (-2.231, -2.073) | -0.621 (-0.829, -0.413) | -0.132 (-0.147, -0.116) |
| Viet Nam | -0.580 (-0.626, -0.534) | -1.826 (-1.891, -1.760) | 0.113 (0.016, 0.209) | -0.123 (-0.139, -0.108) |
| Yemen | -1.806 (-2.242, -1.368) | -1.433 (-1.499, -1.367) | -0.351 (-0.567, -0.135) | -0.157 (-0.171, -0.142) |
| Zambia | -0.771 (-0.855, -0.687) | -1.751 (-1.879, -1.624) | -0.900 (-0.951, -0.849) | -0.059 (-0.081, -0.036) |
| Zimbabwe | -0.266 (-0.468, -0.064) | -1.643 (-1.845, -1.442) | 0.183 (0.085, 0.281) | 0.028 (0.020, 0.037) |

ASIR, age-standardized incidence rate; AVH, acute viral hepatitis; AHA, acute hepatitis A; AHB, acute hepatitis B; AHC, acute hepatitis C; AHE, acute hepatitis E.

**Table S3. Annual percentage change in the incidence of AVH by sex and age from 2000 to 2021**

| **age-male** | **AHA** | **AHB** | **AHC** | **AHE** |
| --- | --- | --- | --- | --- |
| <5 | -0.88 (-0.97, -0.79) | -5.08 (-5.63, -4.54) | -0.70 (-0.76, -0.65) | 0.12 (0.08, 0.16) |
| 5-9 | -0.18 (-0.26, -0.11) | -6.10 (-6.25, -5.95) | -0.00 (-0.04, 0.04) | -0.08 (-0.11, -0.06) |
| 10-14 | -0.50 (-0.59, -0.40) | -5.67 (-6.03, -5.31) | 0.30 (0.25, 0.34) | -0.39 (-0.46, -0.32) |
| 15-19 | -0.71 (-0.82, -0.60) | -3.95 (-4.48, -3.41) | 0.37 (0.30, 0.45) | -0.57 (-0.67, -0.46) |
| 20-24 | -1.36 (-1.55, -1.17) | -1.81 (-2.18, -1.44) | 0.17 (0.03, 0.31) | -0.56 (-0.68, -0.44) |
| 25-29 | -0.89 (-0.99, -0.78) | -1.24 (-1.44, -1.05) | 0.07 (-0.14, 0.28) | -0.52 (-0.58, -0.47) |
| 30-34 | -1.25 (-1.35, -1.15) | -1.41 (-1.56, -1.26) | 0.13 (-0.06, 0.33) | -0.54 (-0.65, -0.42) |
| 35-39 | -1.97 (-2.06, -1.87) | -1.27 (-1.36, -1.18) | 0.17 (0.04, 0.31) | -0.40 (-0.53, -0.27) |
| 40-44 | -2.70 (-2.87, -2.54) | -0.85 (-0.92, -0.78) | 0.09 (-0.12, 0.29) | -0.06 (-0.18, 0.05) |
| 45-49 | -3.34 (-3.48, -3.20) | -0.72 (-0.93, -0.51) | 0.21 (0.08, 0.35) | 0.07 (0.03, 0.10) |
| 50-54 | -3.79 (-4.03, -3.55) | -0.82 (-1.06, -0.58) | -0.06 (-0.20, 0.08) | 0.13 (0.12, 0.15) |
| 55-59 | -5.26 (-5.68, -4.83) | -1.07 (-1.25, -0.89) | -0.53 (-0.84, -0.22) | 0.12 (0.10, 0.15) |
| 60-64 | 0.27 (0.14, 0.40) | -1.02 (-1.13, -0.92) | -0.86 (-1.17, -0.55) | 0.11 (0.08, 0.15) |
| 65-69 | 0.06 (-0.05, 0.18) | -1.11 (-1.27, -0.95) | -0.95 (-1.11, -0.79) | 0.10 (0.07, 0.12) |
| 70-74 | 0.10 (-0.00, 0.20) | -1.02 (-1.10, -0.94) | -0.66 (-0.74, -0.57) | 0.08 (0.05, 0.11) |
| 75-79 | -0.10 (-0.31, 0.12) | -0.90 (-1.01, -0.79) | -0.32 (-0.37, -0.27) | 0.07 (0.05, 0.10) |
| 80-84 | 0.11 (-0.19, 0.41) | -0.93 (-1.09, -0.77) | -0.24 (-0.28, -0.20) | 0.04 (0.01, 0.07) |
| 85-89 | 0.07 (-0.21, 0.36) | -0.88 (-1.05, -0.70) | -0.32 (-0.36, -0.27) | 0.00 (-0.04, 0.04) |
| 90-94 | -0.29 (-0.55, -0.02) | -0.59 (-0.68, -0.51) | -0.44 (-0.51, -0.38) | -0.02 (-0.03, 0.00) |
| 95+ | 2.77 (2.34, 3.19) | -0.46 (-0.58, -0.34) | -0.47 (-0.51, -0.42) | -0.01 (-0.04, 0.01) |
| **age-female** | **AHA** | **AHB** | **AHC** | **AHE** |
| <5 | -1.19 (-1.32, -1.06) | -5.17 (-5.69, -4.64) | -0.70 (-0.81, -0.59) | 0.15 (0.10, 0.19) |
| 5-9 | -0.27 (-0.38, -0.16) | -6.05 (-6.19, -5.91) | -0.22 (-0.38, -0.06) | -0.04 (-0.08, 0.00) |
| 10-14 | -0.50 (-0.62, -0.38) | -5.52 (-5.90, -5.15) | 0.05 (-0.13, 0.23) | -0.29 (-0.35, -0.23) |
| 15-19 | -0.94 (-1.10, -0.79) | -3.92 (-4.42, -3.43) | 0.20 (0.12, 0.28) | -0.45 (-0.53, -0.36) |
| 20-24 | -1.62 (-1.81, -1.44) | -1.82 (-2.21, -1.42) | 0.02 (-0.11, 0.14) | -0.49 (-0.60, -0.37) |
| 25-29 | -0.98 (-1.07, -0.89) | -1.42 (-1.53, -1.31) | -0.08 (-0.32, 0.16) | -0.49 (-0.53, -0.45) |
| 30-34 | -1.15 (-1.20, -1.09) | -1.10 (-1.20, -1.00) | 0.13 (-0.07, 0.33) | -0.53 (-0.64, -0.42) |
| 35-39 | -1.55 (-1.64, -1.46) | -1.13 (-1.19, -1.07) | 0.35 (0.23, 0.46) | -0.41 (-0.54, -0.27) |
| 40-44 | -1.67 (-1.81, -1.52) | -0.79 (-0.88, -0.70) | 0.08 (-0.12, 0.28) | -0.06 (-0.19, 0.06) |
| 45-49 | -1.64 (-1.74, -1.54) | -0.71 (-0.82, -0.60) | -0.12 (-0.33, 0.08) | 0.06 (0.03, 0.09) |
| 50-54 | -2.73 (-3.00, -2.45) | -0.74 (-0.89, -0.59) | -0.44 (-0.59, -0.28) | 0.12 (0.09, 0.15) |
| 55-59 | -4.02 (-4.45, -3.60) | -0.87 (-0.96, -0.78) | -0.82 (-1.12, -0.51) | 0.10 (0.09, 0.12) |
| 60-64 | 0.13 (-0.03, 0.28) | -0.87 (-0.95, -0.79) | -1.07 (-1.38, -0.75) | 0.09 (0.05, 0.12) |
| 65-69 | -0.11 (-0.23, 0.02) | -0.97 (-1.07, -0.87) | -1.06 (-1.23, -0.89) | 0.07 (0.04, 0.09) |
| 70-74 | -0.23 (-0.38, -0.08) | -0.80 (-0.90, -0.71) | -0.64 (-0.74, -0.55) | 0.06 (0.04, 0.08) |
| 75-79 | -0.71 (-0.98, -0.44) | -0.59 (-0.65, -0.52) | -0.17 (-0.22, -0.12) | 0.07 (0.04, 0.10) |
| 80-84 | -0.74 (-1.05, -0.43) | -0.50 (-0.60, -0.39) | -0.04 (-0.08, -0.00) | 0.07 (0.05, 0.10) |
| 85-89 | -0.41 (-0.62, -0.19) | -0.51 (-0.72, -0.31) | -0.09 (-0.12, -0.05) | 0.06 (0.04, 0.08) |
| 90-94 | -0.13 (-0.42, 0.17) | -0.27 (-0.44, -0.11) | -0.14 (-0.22, -0.05) | 0.06 (0.05, 0.07) |
| 95+ | 0.20 (-0.29, 0.69) | -0.03 (-0.08, 0.01) | -0.11 (-0.19, -0.02) | 0.06 (0.05, 0.08) |

AVH, acute viral hepatitis; AHA, acute hepatitis A; AHB, acute hepatitis B; AHC, acute hepatitis C; AHE, acute hepatitis E.

**Table S4. Annual percentage change in ASIR of AVH at the level of sociodemographic index from 2000 to 2021**

| SDI | AHA | AHB | AHC | AHE |
| --- | --- | --- | --- | --- |
| Low | -2.00 (-2.09, -1.91) | -1.33 (-1.40, -1.27) | -0.69 (-0.75, -0.64) | -0.21 (-0.22, -0.19) |
| Low-middle | -2.05 (-2.16, -1.94) | -1.55 (-1.65, -1.46) | -0.26 (-0.36, -0.15) | -0.36 (-0.39, -0.33) |
| Middle | -0.49 (-0.53, -0.45) | -2.61 (-2.69, -2.53) | -0.40 (-0.49, -0.31) | -0.23 (-0.28, -0.17) |
| High-middle | -0.32 (-0.38, -0.26) | -3.43 (-3.53, -3.33) | -0.68 (-0.83, -0.53) | -0.51 (-0.60, -0.42) |
| High | 0.06 (-0.01, 0.13) | -2.54 (-2.65, -2.42) | -0.34 (-0.52, -0.16) | -0.08 (-0.09, -0.06) |

SDI, sociodemographic index; ASIR, age-standardized incidence rate; AVH, acute viral hepatitis; AHA, acute hepatitis A; AHB, acute hepatitis B; AHC, acute hepatitis C; AHE, acute hepatitis E.

**Table S5. Disparity of 204 countries and regions from the frontier ASIR level**

| **location** | **AHA** | **AHB** | **AHC** | **AHE** |
| --- | --- | --- | --- | --- |
| Afghanistan | 1335.95 | 447.51 | 95.33 | 42.6 |
| Albania | 1352.86 | 163.81 | 17.04 | 194.79 |
| Algeria | 984.04 | 545.06 | 66.4 | 79.95 |
| American Samoa | 908.22 | 790.12 | 31.4 | 168.37 |
| Andorra | 350.57 | 114.01 | 26.8 | 28.74 |
| Angola | 472.54 | 2138.42 | 176.66 | 181.17 |
| Antigua and Barbuda | 1229.09 | 84.33 | 36.94 | 194.31 |
| Argentina | 973.4 | 59.57 | 10.39 | 0.66 |
| Armenia | 1051.45 | 352.51 | 148.62 | 161.15 |
| Australia | 605.76 | 294.39 | 58.69 | 48.27 |
| Austria | 481.31 | 85.78 | 15.09 | 29.57 |
| Azerbaijan | 994.18 | 649.79 | 193.41 | 166.39 |
| Bahamas | 1577.95 | 116.69 | 37.49 | 192.99 |
| Bahrain | 1327.46 | 531.61 | 64.88 | 80.29 |
| Bangladesh | 633.13 | 579.68 | 6.85 | 337.34 |
| Barbados | 1208.97 | 63.84 | 33.51 | 190 |
| Belarus | 1086.61 | 19.99 | 62.2 | 91.79 |
| Belgium | 504.56 | 27.82 | 18.56 | 110.14 |
| Belize | 1030.79 | 123.57 | 36.95 | 154.54 |
| Benin | 550.46 | 972.94 | 106.06 | 112.19 |
| Bermuda | 1552.98 | 72.25 | 24.12 | 191.37 |
| Bhutan | 395.26 | 392.72 | 69.49 | 44.39 |
| Bolivia (Plurinational State of) | 1095.54 | 124.29 | 11.99 | 133.48 |
| Bosnia and Herzegovina | 587.45 | 210.24 | 1.75 | 188.92 |
| Botswana | 1220.75 | 733.37 | 77.25 | 167.95 |
| Brazil | 622.42 | 163.61 | 69.17 | 60.33 |
| Brunei Darussalam | 622.97 | 309.28 | 55.24 | 105.1 |
| Bulgaria | 1476.27 | 258.41 | 17.77 | 186.49 |
| Burkina Faso | 168.73 | 1276.71 | 129.59 | 78.62 |
| Burundi | 194.42 | 23.36 | 150.85 | 142.62 |
| C?te d'Ivoire | 552.78 | 1314.29 | 93.33 | 116.79 |
| Cabo Verde | 1065.34 | 1148.43 | 80.39 | 105.34 |
| Cambodia | 317.85 | 924.91 | 140.37 | 92.14 |
| Cameroon | 551.7 | 1246.92 | 116.53 | 113.08 |
| Canada | 349.24 | 305.53 | 14.87 | 49.03 |
| Central African Republic | 49.12 | 1458.86 | 157.48 | 209.67 |
| Chad | 573.77 | 1313.03 | 62.18 | 77.78 |
| Chile | 1268.46 | 117.87 | 28.84 | 39.07 |
| China | 854.64 | 890.39 | 25.67 | 322.85 |
| Colombia | 913.09 | 336.31 | 37.12 | 86.25 |
| Comoros | 712.53 | 711.2 | 79.79 | 190.63 |
| Congo | 1101.5 | 1296.49 | 156.32 | 188.42 |
| Cook Islands | 1056.99 | 404.35 | 26.43 | 166.23 |
| Costa Rica | 1341.65 | 70.45 | 45.54 | 84.13 |
| Croatia | 889.59 | 257.55 | 13.01 | 185.37 |
| Cuba | 853.15 | 0 | 36.42 | 194.75 |
| Cyprus | 301.45 | 105.76 | 10.2 | 29.29 |
| Czechia | 833.1 | 104.87 | 17.56 | 185.82 |
| Democratic People's Republic of Korea | 440.29 | 1753.14 | 41.35 | 182.31 |
| Democratic Republic of the Congo | 425.81 | 2052.38 | 179.66 | 184.66 |
| Denmark | 148.41 | 189.95 | 14.65 | 29.02 |
| Djibouti | 697.68 | 870.32 | 79.5 | 192.04 |
| Dominica | 1372.65 | 135.54 | 36.19 | 196.38 |
| Dominican Republic | 1053.81 | 214.09 | 46 | 154.93 |
| Ecuador | 1087.05 | 151.95 | 21.26 | 179.59 |
| Egypt | 969.27 | 584.85 | 300.09 | 34.29 |
| El Salvador | 1122.48 | 50.4 | 40.81 | 42.77 |
| Equatorial Guinea | 1043.95 | 707.64 | 139.04 | 225.98 |
| Eritrea | 789.02 | 666.32 | 104.47 | 190.68 |
| Estonia | 1161.69 | 75.01 | 60.15 | 90.57 |
| Eswatini | 1261.3 | 822.77 | 70.26 | 124.71 |
| Ethiopia | 678.45 | 737.21 | 95.15 | 234.04 |
| Fiji | 659.67 | 361.94 | 33.87 | 170.12 |
| Finland | 0 | 201.17 | 17.23 | 28.79 |
| France | 551.8 | 73.19 | 11.36 | 19.39 |
| Gabon | 1029.81 | 1073.35 | 200.42 | 198.2 |
| Gambia | 494.36 | 755.78 | 73.86 | 121.44 |
| Georgia | 1228.99 | 276.58 | 150.23 | 164.26 |
| Germany | 341.46 | 18.89 | 16.82 | 28.69 |
| Ghana | 1257.68 | 1513.67 | 122 | 108.33 |
| Greece | 477.15 | 254.43 | 2.48 | 30.61 |
| Greenland | 876.57 | 273.84 | 16.95 | 49.29 |
| Grenada | 870.14 | 108.04 | 42.01 | 195.48 |
| Guam | 1295.38 | 890.89 | 31.35 | 165.36 |
| Guatemala | 1298.25 | 248.88 | 67.7 | 43.99 |
| Guinea | 405.22 | 1590.65 | 117.61 | 120.17 |
| Guinea-Bissau | 867.29 | 1670.18 | 80.14 | 122.04 |
| Guyana | 967.57 | 210.49 | 61.61 | 198.9 |
| Haiti | 498.63 | 275.16 | 65.48 | 151.86 |
| Honduras | 734.97 | 105.41 | 61.06 | 34.55 |
| Hungary | 699.24 | 307.41 | 14.16 | 186.18 |
| Iceland | 29.31 | 125.69 | 7.23 | 28.85 |
| India | 1102.66 | 462.44 | 22.97 | 301.99 |
| Indonesia | 911.91 | 825.97 | 90.02 | 175.56 |
| Iran (Islamic Republic of) | 1068.42 | 312.82 | 30.68 | 125.41 |
| Iraq | 1053.96 | 224.19 | 96.74 | 82.31 |
| Ireland | 491.29 | 88.46 | 15.08 | 29.31 |
| Israel | 497.85 | 2.71 | 7.78 | 20.41 |
| Italy | 808.34 | 143.74 | 48.9 | 53.49 |
| Jamaica | 813.45 | 92.11 | 33.49 | 197.87 |
| Japan | 519.21 | 530.17 | 48.58 | 128.01 |
| Jordan | 1314.2 | 396.5 | 69.63 | 79.46 |
| Kazakhstan | 1090.74 | 205.8 | 100.52 | 165.16 |
| Kenya | 1145.78 | 829.96 | 86.61 | 239.83 |
| Kiribati | 1073.12 | 1382.5 | 59.16 | 131.46 |
| Kuwait | 1635.66 | 101.32 | 49.72 | 78.94 |
| Kyrgyzstan | 1028.04 | 372.05 | 184.13 | 122.75 |
| Lao People's Democratic Republic | 18.25 | 861.97 | 58.82 | 94.59 |
| Latvia | 1228.43 | 0.96 | 48.55 | 91.57 |
| Lebanon | 1169.28 | 528.32 | 37.03 | 61.38 |
| Lesotho | 751.74 | 1079.32 | 78.61 | 120.35 |
| Liberia | 610.52 | 1983.54 | 114.45 | 121.06 |
| Libya | 1232.01 | 424.79 | 59.31 | 81.19 |
| Lithuania | 1224.05 | 34.83 | 69.99 | 91.27 |
| Luxembourg | 351.26 | 84.59 | 16.79 | 29.37 |
| Madagascar | 645.2 | 875.54 | 59.69 | 181.84 |
| Malawi | 687.17 | 419.5 | 102.12 | 165.14 |
| Malaysia | 766.95 | 467.36 | 86.17 | 121.31 |
| Maldives | 517.94 | 289.14 | 83.41 | 127.94 |
| Mali | 43.29 | 580.7 | 33.63 | 75.75 |
| Malta | 516.6 | 11.87 | 8.24 | 29.45 |
| Marshall Islands | 1078.68 | 956.27 | 50.96 | 126.99 |
| Mauritania | 709.19 | 2553.23 | 83.14 | 102.2 |
| Mauritius | 755.28 | 465.03 | 93.33 | 119.67 |
| Mexico | 1070.74 | 0.03 | 43.3 | 130.67 |
| Micronesia (Federated States of) | 71.85 | 861.37 | 47.79 | 128.4 |
| Monaco | 352.51 | 55.79 | 20.13 | 28.84 |
| Mongolia | 1040.86 | 1432.41 | 371.3 | 127.54 |
| Montenegro | 1059.55 | 103.8 | 13.86 | 189.11 |
| Morocco | 992.79 | 351.7 | 61.64 | 35.87 |
| Mozambique | 203.86 | 1052 | 77.98 | 172.92 |
| Myanmar | 575.37 | 378 | 84.98 | 100.99 |
| Namibia | 1225.12 | 1086.16 | 62.54 | 124.29 |
| Nauru | 643.57 | 725.67 | 57.33 | 125.31 |
| Nepal | 480.29 | 278.17 | 7.84 | 184.52 |
| Netherlands | 350.58 | 44.03 | 3.83 | 20.22 |
| New Zealand | 469.05 | 226.38 | 32.45 | 65.11 |
| Nicaragua | 1078.06 | 0 | 68.72 | 42.44 |
| Niger | 192.3 | 499.72 | 0.69 | 2.25 |
| Nigeria | 540.98 | 2282.82 | 125.49 | 152.09 |
| Niue | 913.58 | 386.82 | 36.74 | 168.18 |
| North Macedonia | 873.71 | 227.18 | 17.77 | 187.46 |
| Northern Mariana Islands | 1057.76 | 1377.68 | 43.02 | 166.29 |
| Norway | 426.36 | 43.65 | 1.11 | 41.69 |
| Oman | 1532.07 | 433.23 | 62.52 | 80.53 |
| Pakistan | 756.54 | 516.35 | 101.42 | 224.31 |
| Palau | 1135.17 | 695.22 | 45.76 | 168.1 |
| Palestine | 1129.4 | 471.47 | 73.24 | 49.71 |
| Panama | 1403.18 | 27.02 | 35.1 | 87.06 |
| Papua New Guinea | 344.23 | 1321.87 | 55.36 | 148.05 |
| Paraguay | 584.09 | 217.62 | 58.39 | 12.43 |
| Peru | 1073.71 | 351.55 | 18.04 | 180.59 |
| Philippines | 496.87 | 1336.53 | 59.08 | 178.66 |
| Poland | 999.37 | 42.99 | 14.61 | 224.62 |
| Portugal | 195.51 | 67.65 | 34.36 | 27.62 |
| Puerto Rico | 1554.49 | 52.5 | 34.62 | 190.65 |
| Qatar | 1644.54 | 520.44 | 69.32 | 79.75 |
| Republic of Korea | 945.01 | 667.75 | 35.82 | 70.49 |
| Republic of Moldova | 960.34 | 416.31 | 92.91 | 196.71 |
| Romania | 733.02 | 384.13 | 33.11 | 190.85 |
| Russian Federation | 1375.21 | 107.22 | 66.32 | 56.61 |
| Rwanda | 736.75 | 492.39 | 97.19 | 165.48 |
| Saint Kitts and Nevis | 1276.41 | 127.43 | 39.74 | 193.17 |
| Saint Lucia | 872.13 | 143.99 | 42.18 | 195.73 |
| Saint Vincent and the Grenadines | 923.58 | 106.6 | 39.67 | 165.17 |
| Samoa | 583.18 | 1102 | 45.25 | 119.65 |
| San Marino | 354.91 | 71.61 | 16.01 | 9.89 |
| Sao Tome and Principe | 531.72 | 1769.09 | 93.59 | 98.64 |
| Saudi Arabia | 1413.84 | 370.6 | 130.07 | 150.75 |
| Senegal | 552.67 | 1362.96 | 66.84 | 103.77 |
| Serbia | 1524.11 | 152.28 | 16.94 | 187.06 |
| Seychelles | 767.11 | 509.3 | 66.28 | 125.87 |
| Sierra Leone | 625.56 | 1030.87 | 102.34 | 139.08 |
| Singapore | 608.24 | 510.78 | 55.61 | 152.27 |
| Slovakia | 1149.14 | 195.79 | 17.18 | 185.45 |
| Slovenia | 1144.66 | 208.82 | 15.26 | 185.17 |
| Solomon Islands | 160.75 | 1020.38 | 55.83 | 122.13 |
| Somalia | 0 | 22.02 | 0.04 | 2.11 |
| South Africa | 1074.51 | 266.05 | 60.11 | 200.28 |
| South Sudan | 338.61 | 784.65 | 73.01 | 148.3 |
| Spain | 249.31 | 225.58 | 21.58 | 20.2 |
| Sri Lanka | 339.21 | 514.27 | 62.41 | 144.04 |
| Sudan | 1358.93 | 1119.99 | 68.06 | 51.7 |
| Suriname | 928.99 | 122.3 | 48.69 | 164.15 |
| Sweden | 67.38 | 63.2 | 2.71 | 41.91 |
| Switzerland | 245.52 | 159.37 | 20.38 | 39.17 |
| Syrian Arab Republic | 1026.87 | 702.95 | 77.04 | 35.07 |
| Taiwan (Province of China) | 1077.66 | 908.41 | 79.52 | 148.57 |
| Tajikistan | 1152.38 | 400.25 | 189.11 | 139.6 |
| Thailand | 336.84 | 572.81 | 59.1 | 128.96 |
| Timor-Leste | 1.48 | 709.89 | 58.44 | 94.07 |
| Togo | 514.33 | 1469.16 | 98.05 | 114.3 |
| Tokelau | 636.77 | 806.26 | 42.94 | 169.1 |
| Tonga | 853.2 | 962.24 | 66.03 | 137.75 |
| Trinidad and Tobago | 1262.6 | 121.55 | 34.83 | 194.05 |
| Tunisia | 991.28 | 367.51 | 69.55 | 63.67 |
| Türkiye | 1024.97 | 420.49 | 29.64 | 93.23 |
| Turkmenistan | 1011.85 | 429.78 | 212.32 | 168.36 |
| Tuvalu | 663.31 | 1137.91 | 44.62 | 124.5 |
| Uganda | 741.09 | 891.52 | 111.14 | 186.39 |
| Ukraine | 908.93 | 120.38 | 101.99 | 119.71 |
| United Arab Emirates | 1643.56 | 151.66 | 54.91 | 80.12 |
| United Kingdom | 470.39 | 158 | 22.74 | 40.14 |
| United Republic of Tanzania | 675.47 | 758.7 | 137.06 | 166.27 |
| United States of America | 638.97 | 0 | 24.26 | 64.89 |
| United States Virgin Islands | 1560.77 | 61.85 | 37.39 | 192.57 |
| Uruguay | 969.54 | 21.85 | 11.36 | 32.51 |
| Uzbekistan | 1144.88 | 981.13 | 206.1 | 168.62 |
| Vanuatu | 185.96 | 1178.57 | 57.3 | 126.22 |
| Venezuela (Bolivarian Republic of) | 1047.58 | 64.31 | 23.64 | 22.74 |
| Viet Nam | 704.33 | 1487.63 | 63.77 | 110.75 |
| Yemen | 602.1 | 1150.01 | 93.95 | 0 |
| Zambia | 738.95 | 878.11 | 100.17 | 165.2 |
| Zimbabwe | 868.17 | 2525.05 | 88.55 | 118.57 |

AVH, acute viral hepatitis; AHA, acute hepatitis A; AHB, acute hepatitis B; AHC, acute hepatitis C; AHE, acute hepatitis E.

**Table S6. BAPC model predicts the incidence and ASIR of AVH over the next 15 years**

| **Number** | **AHA** | **AHB** | **AHC** | **AHE** |
| --- | --- | --- | --- | --- |
| 2022 | 162437322 | 63714593 | 7082570 | 19620465 |
| 2023 | 161406254 | 62697799 | 7134253 | 19746219 |
| 2024 | 160281330 | 61767713 | 7186033 | 19865202 |
| 2025 | 159083440 | 60960044 | 7237024 | 19978496 |
| 2026 | 157847771 | 60177762 | 7288024 | 20088456 |
| 2027 | 156591426 | 59256405 | 7338582 | 20195148 |
| 2028 | 155353321 | 58265384 | 7391127 | 20300716 |
| 2029 | 154113739 | 57386709 | 7445369 | 20400618 |
| 2030 | 152900524 | 56664671 | 7500731 | 20496029 |
| 2031 | 151739418 | 55997118 | 7558319 | 20589559 |
| 2032 | 150639902 | 55222105 | 7617967 | 20681273 |
| 2033 | 149612028 | 54401331 | 7680563 | 20774066 |
| 2034 | 148648990 | 53711672 | 7746050 | 20864696 |
| 2035 | 147770427 | 53201260 | 7813754 | 20954471 |
| 2036 | 146991898 | 52772376 | 7884874 | 21045394 |
| **ASIR** | **AHA** | **AHB** | **AHC** | **AHE** |
| 2022 | 2240.74 | 768.49 | 91.02 | 261.28 |
| 2023 | 2220.67 | 748.23 | 90.76 | 261.69 |
| 2024 | 2200.29 | 729.53 | 90.5 | 262.09 |
| 2025 | 2179.7 | 712.79 | 90.24 | 262.5 |
| 2026 | 2158.98 | 696.78 | 90 | 262.93 |
| 2027 | 2138.14 | 679.36 | 89.75 | 263.39 |
| 2028 | 2117.15 | 661.42 | 89.51 | 263.86 |
| 2029 | 2096.05 | 645.2 | 89.27 | 264.31 |
| 2030 | 2074.9 | 631.2 | 89.02 | 264.77 |
| 2031 | 2053.76 | 618.19 | 88.78 | 265.24 |
| 2032 | 2032.63 | 604.15 | 88.55 | 265.73 |
| 2033 | 2011.47 | 589.82 | 88.31 | 266.22 |
| 2034 | 1990.32 | 577.25 | 88.08 | 266.71 |
| 2035 | 1969.22 | 566.97 | 87.84 | 267.2 |
| 2036 | 1948.21 | 557.85 | 87.6 | 267.7 |

ASIR, Age-standardized incidence rate; AVH, acute viral hepatitis; AHA, acute hepatitis A; AHB, acute hepatitis B; AHC, acute hepatitis C; AHE, acute hepatitis E; BAPC, Bayesian age-period-cohort model.
